# A novel CRISPR-engineered prostate cancer cell line defines the AR-V transcriptome and identifies PARP inhibitor sensitivities

**DOI:** 10.1093/nar/gkz286

**Published:** 2019-04-22

**Authors:** Evangelia Kounatidou, Sirintra Nakjang, Stuart R C McCracken, Scott M Dehm, Craig N Robson, Dominic Jones, Luke Gaughan

**Affiliations:** 1 Northern Institute for Cancer Research, Newcastle University, Paul O’Gorman Building, Framlington Place, Newcastle Upon Tyne, NE2 4HH, UK; 2 University of Minnesota, Department of Laboratory Medicine and Pathology, MMC 806 Mayo, 420 Delaware, Minneapolis, MN 55455, USA

## Abstract

Resistance to androgen receptor (AR)-targeted therapies in prostate cancer (PC) is a major clinical problem. A key mechanism of treatment resistance in advanced PC is the generation of alternatively spliced forms of the AR termed AR variants (AR-Vs) that are refractory to targeted agents and drive tumour progression. Our understanding of how AR-Vs function is limited due to difficulties in distinguishing their discriminate activities from full-length AR (FL-AR). Here we report the development of a novel CRISPR-derived cell line which is a derivative of CWR22Rv1 cells, called CWR22Rv1-AR-EK, that has lost expression of FL-AR, but retains all endogenous AR-Vs. From this, we show that AR-Vs act unhindered by loss of FL-AR to drive cell growth and expression of androgenic genes. Global transcriptomics demonstrate that AR-Vs drive expression of a cohort of DNA damage response genes and depletion of AR-Vs sensitises cells to ionising radiation. Moreover, we demonstrate that AR-Vs interact with PARP1 and PARP2 and are dependent upon their catalytic function for transcriptional activation. Importantly, PARP blockade compromises expression of AR-V-target genes and reduces growth of CRPC cell lines suggesting a synthetic lethality relationship between AR-Vs and PARP, advocating the use of PARP inhibitors in AR-V positive PC.

## INTRODUCTION

Prostate cancer (PC) is the second most common malignancy in men with approximately 1.3 million new cases reported worldwide in 2018 (World Cancer Research Fund). At presentation, PC growth is androgen-dependent hence current treatments act to attenuate the androgen receptor (AR) signalling axis via the use of hormonal therapy, including anti-androgens (1–3). Although initially successful, patients invariably become resistant to treatment and develop a more aggressive form of the disease termed castrate-resistant PC (CRPC) which, in most cases, remains dependent on AR signalling for growth (1,4,5). Typically, persistent AR function is expedited by several molecular alterations, including amplification and mutation of the *AR* gene (6–11), as well as the generation of alternatively spliced variants of the full-length AR (FL-AR), termed AR-Vs (12,13), which enable constitutive androgenic signalling in castrate conditions to drive progression to CRPC.

Critically, AR-Vs represent a major clinical challenge. Unlike wild-type and mutant FL-AR isoforms that are generally repressed by next-generation anti-androgens enzalutamide and apalutamide (14,15), AR-Vs lack the site of targeted therapeutics, but retain conventional N-terminal transactivation and DNA-binding capabilities hence facilitate CRPC progression unchallenged by the current repertoire of receptor-targeting agents (16–18). Importantly, overexpression of a number of AR-Vs, including AR-V7 and AR-V3, has been reported in 20–40% of CRPC patients, with the figure rising further in metastatic disease (18,19). Difficulties in identifying tractable sites within the inherently unstructured N-terminus (20,21) and the challenge of developing selective agents for inactivating AR DNA binding, advocate more research into the regulatory processes that govern AR-V activity in CRPC as a means of identifying and exploiting new therapeutic targets in advanced disease.

A major limitation in the study of AR-V biology, however, is the paucity of models that permit discriminate AR-V-specific functional and phenotypic read-outs that are not influenced by FL-AR. Utilising either FL-AR siRNA-mediated knockdown or enzalutamide treatment in FL-AR- and AR-V-expressing CWR22Rv1 and VCaP cell lines, several groups have attempted to establish models for interrogating splice variant transcriptomics and co-regulator requirements (22,23). Although useful, incomplete FL-AR depletion or anti-androgen-mediated inactivation in these systems is likely to compromise read-outs believed to be AR-V specific and may be a contributing factor to the controversy regarding whether FL-AR and AR-Vs have distinct transcriptional programmes (24). Recently, the development of a TALEN-based genome-edited derivative of the CWR-AD1 cell line, named R1-D567 that expresses the clinically-relevant AR-v567es receptor variant has provided an important extrapolation to our understanding of AR-V-driven transcriptomics and drug sensitivities (25). However, given that multiple AR-Vs have been detected in individual circulating CRPC tumour cells (18,19), consistent with the CWR22Rv1 and VCaP cell lines, there remains a requirement to develop additional clinically-relevant models that express multiple AR-Vs in the absence of FL-AR to enable more robust studies of AR-V biology in advanced disease.

To this end, we have developed the first of its kind CRISPR-derived FL-AR knockout CWR22Rv1 cell line that retains expression of all endogenous AR-Vs making it a valuable model for the study of receptor splice variants. This new derivative called CWR22Rv1-AR-EK (Exon Knockout) is dependent upon AR-Vs for growth, is refractory to all FL-AR-targeting agents and displays a gene expression programme similar to parental CWR22Rv1 cells consistent with FL-AR and AR-V transcriptional mimicry. Furthermore, we demonstrate for the first time that AR-Vs regulate a DNA damage response (DDR) gene network encompassing a FL-AR-like ‘BRCAness’ signature (26), which is critical for cell survival upon ionising radiation treatment. Finally, we provide evidence of a feed-forward regulatory loop between AR-Vs and PARP by demonstrating that AR-Vs (i) are dependent upon PARP activity for transcriptional function, and (ii) enhance expression of *PARPBP* and *PARP2* to upregulate cellular PARP activity. Critically, in the context of AR-V-expressing CRPC, we show that PARP inhibition down-regulates both androgenic and DDR gene expression signatures to attenuate cell proliferation and potentiate a synthetic lethality phenotype indicating clinically-relevant sensitivities in the advanced disease setting.

## MATERIALS AND METHODS

### CRISPR knock-in pipeline and generation of CWR22Rv1-AR-EK cells

Two custom gRNAs were designed to target distinct loci within exon 5 of the *AR* gene (sequences shown in Supplementary Figure S1B) (Sigma). These were cloned into the all-in-one pLenti CRISPR/Cas9 vector (pLV-U6g-EPCG) (Sigma) to generate the Cas9/gRNA_1 and Cas9/gRNA_2 constructs. 2 × 10^6^ CWR22Rv1 cells were transiently nucleofected with 6 μg of Cas9/gRNA_1 or Cas9/gRNA_2 plasmid vector using the Amaxa™ Cell Line Nucleofector™ kit R (Lonza) and the Nucleofector^®^ II device (programme T-009) according to manufacturer's instructions. Editing efficiency of the two distinct CRISPR complexes was assessed in CWR22Rv1 cells using the SURVEYOR assay (Integrated DNA Technologies) according to manufacturer's instructions. This methodology enables the efficiency of CRISPR-mediated DNA insertions and deletions (indels) to be estimated by firstly mixing (at 1:1 ratio) wild-type and genome-edited amplicons, derived from PCR amplification of the target locus, and then digesting mis-annealed duplexes using the Surveyor nuclease. Resultant DNA is then subject to electrophoresis and CRISPR efficiency is calculated by comparing relative intensities of the wild-type amplicon to CRISPR-derived lower molecular weight species using ImageJ. Additionally, PCR products were sequenced and chromatograms were analysed by the TIDE (Tracking of Indels by DEcomposition) algorithm (http://tide-calculator.nki.nl/, Netherlands Cancer Institute) to accurately calculate the editing efficiency of Cas9.

To knock-in a stop codon into exon 5 of the AR locus, a 180 bp ssODN template was custom designed (Sigma) containing a central TAA sequence and flanked by 75 bp 5′ and 3′ termini 100% complementary to the AR gene sequence. Additionally, the stop codon generated an *Mse* I restriction enzyme site which was used in restriction fragment length polymorphism (RFLP)-based analysis to enable detection of successful knock-in clones. CWR22Rv1 cells were co-nucleofected with the donor template (1 μg) and Cas9/gRNA_2 construct (6 μg) for 48 h prior to puromycin selection (2 μg/ml) for 5 days and subsequent single cell sorting using a FACS Aria II flow cytometer (BD Biosciences) to clone out single, DAPI negative cells.

### Cell lines and reagents

LNCaP, CWR22Rv1 and VCaP cells were all purchased from ATCC and authenticated prior to conducting the study (see Supplementary Figure S3B for example of authentication). R1-D567 is a TALEN-engineered cell line derivative of the AD-1 cell line that expresses only the AR-V567es receptor isoform^1^. All cells were maintained in RPMI 1640 media (Sigma) supplemented with 10% foetal calf serum (FCS) and 5% l-glutamine at 37°C and subject to regular mycoplasma testing. Enzalutamide (Selleckchem) and dihydrotestosterone (DHT) (Sigma) were utilised at 10 μM and 10 nM, respectively. PARP inhibitors rucaparib, olaparib and talazoparib were all purchased from Selleckchem and used at 0.5 and 1 μM for assay-specific durations.

### Quantitative PCR and western blot analysis

Quantitative PCR (qPCR) was used to assess expression of candidate AR-V target genes and those identified from RNA sequencing experiments (see Supplementary Table S5 for primer sequences) using cDNA generated from Ribozol (VWR)-mediated RNA extractions as described (27). RNA purity and concentration were calculated using NanoDrop 2000 (Thermo Fischer Scientific). 1μg of RNA was subject to cDNA conversion using the M-MLV reverse transcription kit (Promega) following manufacturer's protocol. Quantitative PCR analysis was performed using the Platinum SYBR Green qPCR SuperMix (Invitrogen) and amplification reactions were carried out on a QuantStudio 7 Flex Real-Time PCR machine (Applied Biosystems) performing the following thermal profile: 50°C for 2 min (UNG activation), 95°C for 10 min (initial denaturation), 95°C for 15 s and 60°C for 1 min (40 cycles) followed by melt curve analysis to assess non-specific amplification. The QuantStudio Real-Time PCR software (Applied Biosystems) was used for data analysis. Standard curve quantification analysis was performed to quantify targets of interest. All quantities determined for samples are, therefore, relative to the quantity assigned to the standard curve which was generated by serial dilutions of the siScr/DMSO treated cDNA sample. Quantity mean values for each target of interest were normalised to the reference housekeeping gene HPRT1 and resultant normalised values were expressed relative to the siSCR/DMSO treated sample depending on the experiment. Data represents the mean of three independent experiments.

Western blotting was performed as described in (28) using the following antibodies: AR(N) (N-20; sc-816), AR(C) (C-19; sc-815), AR-441 (sc-7305) and PARP-1/2 (sc-7150)(all Santa Cruz Biotechnology), Cas9 (ab191468), AR (Ab74272), AR-V7 (ab198394), Histone H2B (ab134211), β-actin (ab49900) and phospho-ATM (ab81292)(all Abcam); α-Tubulin (B-5–1-2)(Sigma); PAR (10H; Enzo Life Sciences); total ATM (D2E2) (Cell Signaling); AR-BD (BD Pharmingen). Antibodies were used at 1:1000 dilution in 5% non-fat skimmed milk (Marvel). Overnight incubations were performed at 4°C. Secondary HRP-conjugated polyclonal rabbit anti-mouse (P0260) and swine anti-rabbit (P0217) antibodies (Dako) were used at 1:1000 for 1 h at room temperature.

### siRNAs and lentiviral transduction

Transient transfection of siRNA to deplete CWR22Rv1-AR-EK and CWR22Rv1 cells of all AR isoforms or discriminately of FL-AR and AR-Vs was performed in dextran-coated charcoal-stripped foetal bovine serum (HyClone) containing media (hereafter called steroid-depleted media) in six-well plates (Corning) using 25nM of siRNA per well and Lipofectamine RNAiMax reagent (Life Sciences) according to manufacturer's instructions. All siRNAs were purchased from Sigma and sequences are listed in Supplementary Table S6. The duration of knockdown was dependent upon experimental readout: for AR-target gene expression analysis knockdown was carried out for 48 h; cell proliferation assays for 96 h; and clonogenic experiments for 2 weeks. LNCaP cells were stably transduced with pLV-AR-V7 derived lentiviral particles for 24 h in steroid-depleted media before being treated with 1 μM talazoparib for an additional 24 h.

### Chromatin immunoprecipitation (ChIP) and immunoprecipitation

ChIP assays were performed as described in (22) utilising AR (N-20), AR (C-19) and PARP1/2 (Santa Cruz Biotechnology) and AR (Cell Signalling) antibodies. Quantitative PCR of resultant immunoprecipitated DNA was performed using primers to *cis*-regulatory elements of AR target genes (see Supplementary Table S3 for sequences). For ChIP experiments investigating recruitment of AR and PARP1/2 to target genes in response to PARP blockade, CWR22Rv1-AR-EK and CWR22Rv1 cells grown in steroid-depleted media were treated with and without 1 μM talazoparib for 4 and 8 h prior to chromatin preparation. For experiments assessing impact of FL-AR and AR-V-targeting siRNAs on AR isoform chromatin enrichment, CWR22Rv1 and CWR22Rv1-AR-EK cells grown in steroid-depleted media were transiently transfected with specific AR siRNAs for 48 hours before ChIP analysis. ChIP data is presented as the mean of at least two independent experiments (±SD). Primers for quantitative *cis*-regulatory element enrichment is shown in Supplementary Table S5.

Immunoprecipitation was conducted as described in (27) using 5 × 10^6^ CWR22Rv1-AR-EK or CWR22Rv1 cells incorporating either the AR 441 or AR C19 antibodies.

### Cell proliferation, clonogenics and immunofluorescence (IF)

CWR22Rv1 and CWR22Rv1-AR-EK cells grown in steroid-depleted conditions and transiently transfected with AR-targeting siRNAs or treated with PARP1/2 inhibitors olaparib and talazoparib for 96 hours were counted using Sulforhodamine B (SRB) assays (as described in (29)) or trypsinised and counted individually using a haemocytometer. Data represents three independent experiments performed in triplicate ± SD. For clonogenic experiments, CWR22Rv1-AR-EK cells were transiently transfected with either control or AR-targeting siRNAs for 48 h prior to re-seeding at densities of 500 and 1000 cells/well in six-well plates (Corning) for two weeks. Colonies were fixed with 10% neutral buffered formalin solution (Sigma) and subsequently stained using 0.01% (w/v) crystal violet before counting using an automated colony counter. To assess the effect of AR-V depletion on sensitivity of cells to ionizing radiation (IR), cells were transiently transfected as described, and 48 h later subject to 2 Gy IR treatment before re-seeding, incubating and quantifying viable cells as before.

For GFP-based IF to detect expression of Cas9/gRNA complexes, 2 × 10^5^ cells grown on glass coverslips in six-well plates were transiently transfected with 3 μg of the appropriate pLV-U6g-EPCG vector for 24 h prior to fixing in 4% paraformaldehyde and mounting cells on glass slides using DAPI-containing mounting media (Vectashield) prior to fluorescence microscopy (Nikon TE2000). AR-V localisation was assessed in CWR22Rv1-AR-EK cells seeded in steroid-depleted media in chamber slides (4000 cells/chamber) (Thermo Fischer Scientific). Staining was carried out using the AR (D6F11) primary antibody (Cell Signaling) at 1:1000 for 1 h at room temperature. Following three PBS washes, cells were incubated with an AlexaFluor 488 secondary antibody for 1 h at room temperature prior to mounting with DAPI and imaging. Finally, to assess γH2AX foci formation after 2 Gy ionising radiation treatment, cells grown on glass coverslips were fixed in 4% paraformaldehyde for 30 min, permeabilized in 0.1% Trinton X-100/PBS for 10 min and then blocked in 4% BSA (Sigma) for 1 h prior to incubation with γH2AX antibody (JBW301, Millipore) at 1:1000 dilution at 4°C overnight. Cells were washed in PBS and then incubated with an AlexaFluor 546 secondary antibody (Life Technologies) for 1 hour prior to mounting. Images were captured as z-stacks using a Leica upright DM6 fluorescence microscope. Automated foci analysis was performed using a macro in ImageJ as described in (30).

### RNA sequencing and transcriptomic analysis

CWR22Rv1-AR-EK cells were transiently transfected in triplicate for 48 hours with control or AR-targeting siRNAs before RNA extraction using RNeasy Mini Kit according to manufacturer's instructions (Qiagen) and QC. 500 ng RNA of each triplicate sample was subject to library preparation using TruSeq Standed mRNA library prep kit (Illumina)(performed by Otogenetics Corporation, Atlanta, USA). Resultant libraries were subject to paired-end (100–125 bp) sequencing on an Illumina HiSeq 2500 sequencer, generating an average of 30 million reads per sample. Each data-set was mapped against the human reference genome (Hg19) utilising STAR and then analysed with HTSeq to extract counts and DESeq2 to perform the differential gene expression comparisons between control and AR-depleted samples utilizing the www.DNAnexus.com portal. Differentially-expressed genes were annotated using a fold change threshold of 1.5 between control and AR-V knockdown arms. False discovery rate (FDR) threshold was set at 0.01; hence up- and down-regulated genes were identified as those with FDR ≤ 0.01 and respective FC of ≥1.5 and ≤–1.5.

Significantly altered genes between control and AR-V-depleted samples identified by RNA sequencing were clustered in function-related gene groups using the Functional Enrichment analysis tool FunRich (31). Expression analysis of DDR genes was performed for BPH (*n* = 12), localized PC (*n* = 49) and metastatic CRPC (*n* = 27) using the Gene Expression Microarray Analysis dataset from Grasso *et al.* (32) (GSE35988). TCGA-PRAD gene expression data were downloaded from The Genomic Data Commons (GDC) (33) legacy database and normalised. Differential expression analysis was performed using Bioconductor TCGAbiolinks package (version 2.9.0) (34). In brief, after excluding samples without AR-V7 status, a total of 333 samples remained (249 AR-V7 absence and 84 AR-V7 presence) (35). AR-V7 was determined as present in a sample if at least two splice reads were identified spanning the 3′ end of exon 3 and the 5′ end of the downstream cryptic exon, with a minimum of 6nt overhang on either side without mismatches. Raw counts were extracted and were normalised using the gcContent method. Differential expression analysis was performed using the glmLRT method.

### Statistics

Unless stated otherwise, graphical data shown in each figure represents the mean of three independent experiments and error bars indicate ± standard deviation (SD). For analyzing the effect of siRNA-mediated knockdown or PARP inhibitor treatment on AR-mediated gene expression, chromatin enrichment and cell viability by qRT-PCR, ChIP and clonogenics experiments, respectively, one-way ANOVA and two-tailed student T-tests were conducted depending on the number of variables and *P < 0.05, **P < 0.01, ***P < 0.001 and ****P < 0.0001 were classified as statistically significant. For analysis of γH2AX foci, a Mann–Whitney test was applied.

## RESULTS

### Development of the CWR22Rv1-AR-EK cell line

**Figure 1.**
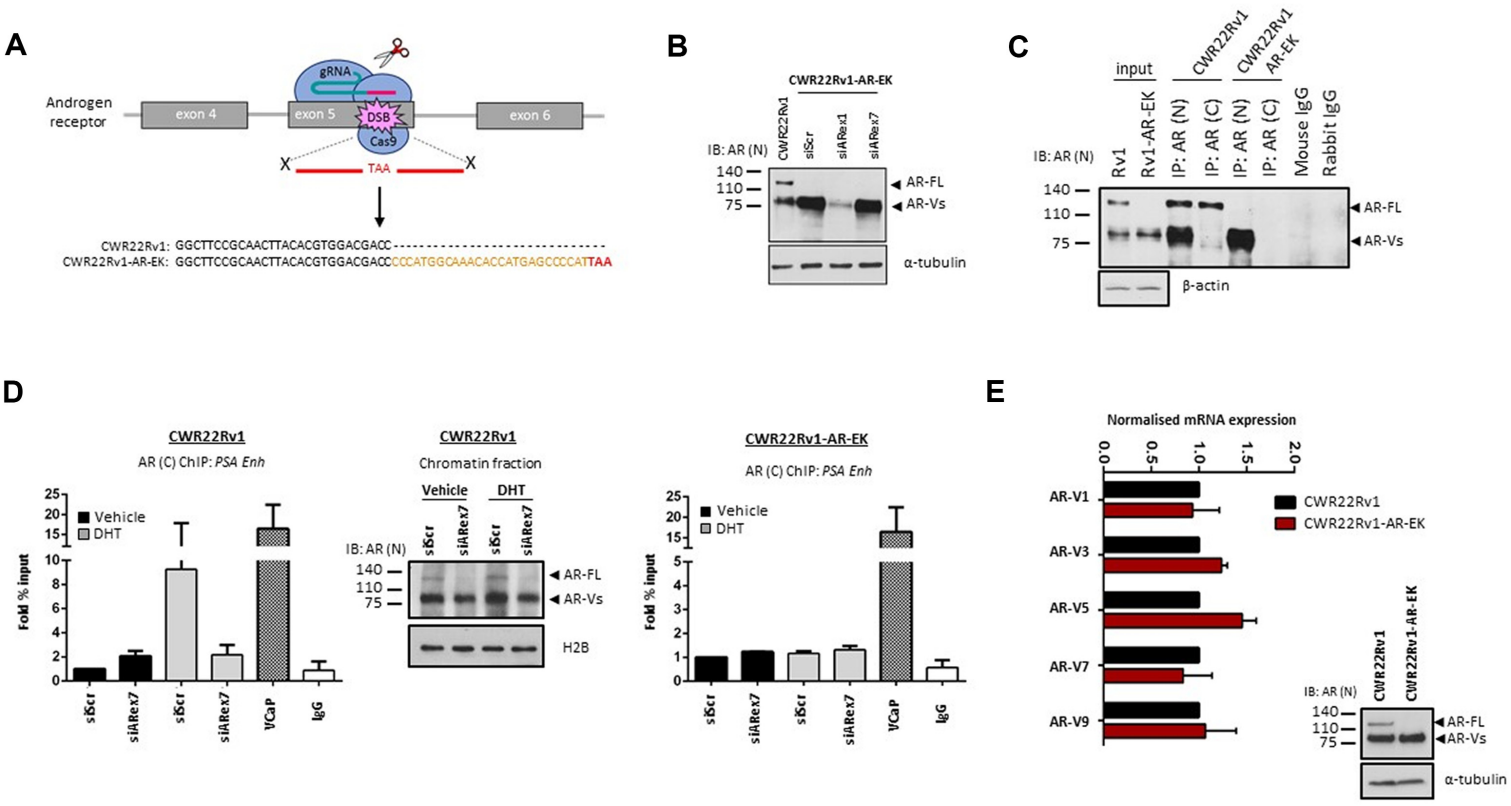
Development and validation of the CWR22Rv1-AR-EK cell line. (**A**) Diagrammatic representation of the CRISPR strategy utilised to introduce a stop codon into the FL-AR-encoding exon 5 of the *AR* gene. Sequence of parental and CWR22Rv1-AR-EK AR locus adjacent to PAM site of Cas9/gRNA_2 is shown. (**B)** Western blotting of either parental CWR22Rv1 cells or CWR22Rv1-AR-EK cells subject to control (siScr), N-terminal AR-targeting (siARex1) or C-terminal-targeting (siARex7) siRNAs for 48 hours, using an N-terminal-binding AR antibody and α-tubulin for loading control. (**C**) CWR22Rv1 and CWR22Rv1-AR-EK cells were subject to immunoprecipitation (IP) incorporating either N- or C-terminal-binding AR antibodies and resultant immunoprecipitates probed with an N-terminal AR-binding antibody. Input samples were ran alongside IP samples and additionally probed with α-tubulin to demonstrate parity in protein quantities between the IP experimental arms. (**D**) CWR22Rv1 (left panel) and CWR22Rv1-AR-EK (right panel) cells grown in steroid-depleted media supplemented with and without 10 nM dihydrotestosterone (DHT) were subject to either siScr or siARex7 transfection for 48 hours prior to chromatin immunoprecipitation (ChIP) using C-terminal AR-binding or control (IgG) antibodies and quantitative PCR incorporating primers to the *PSA* enhancer. VCaP cells treated with 10 nM DHT for 4 hours were used as a positive control for enrichment of FL-AR. Data represents the average of three independent experiments ± SD. Validation of siRNA-mediated FL-AR knockdown was demonstrated by western blotting of CWR22Rv1 chromatin fractions incorporating anti-AR and histone H2B antibodies. (**E**) Quantitative RT-PCR to compare expression of clinically-relevant AR-Vs in CWR22Rv1 and CWR22Rv1-AR-EK cells grown in serum-containing media was performed. Data represents the average of three independent experiments ± SD.

We developed a CRISPR/Cas9 knock-in strategy in CWR22Rv1 cells to introduce a translational stop codon downstream of the AR DBD in exon 5 of the *AR* gene to ablate cellular FL-AR levels while maintaining expression of all AR-Vs endogenous to the parental cell line (Figure 1A). Two specific guide RNAs (gRNAs) were designed to the desired locus, co-expressed with Cas9 and the cleavage efficiency was determined using both SURVEYOR endonuclease and TIDE analysis in CWR22Rv1 cells (36) (Supplementary Figure S1A and B). The more efficient Cas9/gRNA_2 complex was utilized in conjunction with a single-stranded donor DNA template containing the desired stop codon; which also doubled as an *Mse* I restriction site for downstream restriction fragment length polymorphism (RFLP) analysis (Supplementary Figure S1C) to facilitate detection of genome-edited clones in CWR22Rv1 cells. Successful incorporation of the donor template was detected as evidenced by two cleaved DNA products in the RFLP assay (Supplementary Figure S1D) and the correct reading-frame was confirmed by sequencing of the target *AR* gene locus (data not shown). This CWR22Rv1 cell derivative was named CWR22Rv1-AR-EK (Exon Knockout) and, as predicted, FL-AR was not detectable by western blot analysis utilizing multiple N-terminal-binding antibodies, but AR-V levels were maintained and could be depleted using exon 1-targeting siRNAs (Figure 1B and Supplementary Figure S2). To further validate loss of FL-AR in the CWR22Rv1-AR-EK cell line, we show that in contrast to CWR22Rv1 parental cells, no FL-AR was immunoprecipitated using a C-terminal epitope-targeting AR antibody in the CWR22Rv1-AR-EK cell line; and only AR-Vs, but not FL-AR, were immunoprecipitated using an anti-N-terminal AR antibody (Figure 1C). Given that the stop codon was introduced in exon 5, there is the potential to generate an additional AR derivative consisting of *AR* exons 1–4 which would be approximately 9 amino acids larger than the numerous endogenous AR-Vs. Interrogation of western blotting data (Figure 1B, C and Supplementary Figure S2) shows that no additional AR species are generated by this CRISPR strategy. In chromatin immunoprecipitation (ChIP) experiments, successful dihydrotestosterone (DHT)-induced enrichment of FL-AR to the *PSA* gene enhancer in CWR22Rv1 cells using a C-terminal AR-binding antibody, that was attenuated by an AR exon 7-targeting siRNA (Figure 1D, left and middle panels), could not be replicated in the CWR22Rv1-AR-EK derivative (Figure 1D, right panel) validating that FL-AR has been lost from this cell line. Importantly, expression of all AR-Vs endogenous to parental CWR22Rv1 were unchanged in the genome-edited derivative (Figure 1E). From a morphological and karyotype perspective, both CWR22Rv1 parental and –AR-EK derivative are equivalent (Supplementary Figure S3A and B) and the top-ranked predicted exonic Cas9/gRNA_2 off-target loci (as determined using CCTop; https://crispr.cos.uni-heidelberg.de/ and CRISPR design tool crispr.mit.edu) were sequenced and showed no mutations compared to wild-type suggesting that any alteration to this cell line is a consequence of FL-AR loss and not aberrant CRISPR-mediated mutations (Supplementary Figure S3C).

### AR-Vs maintain expression of AR target genes in the absence of FL-AR

**Figure 2.**
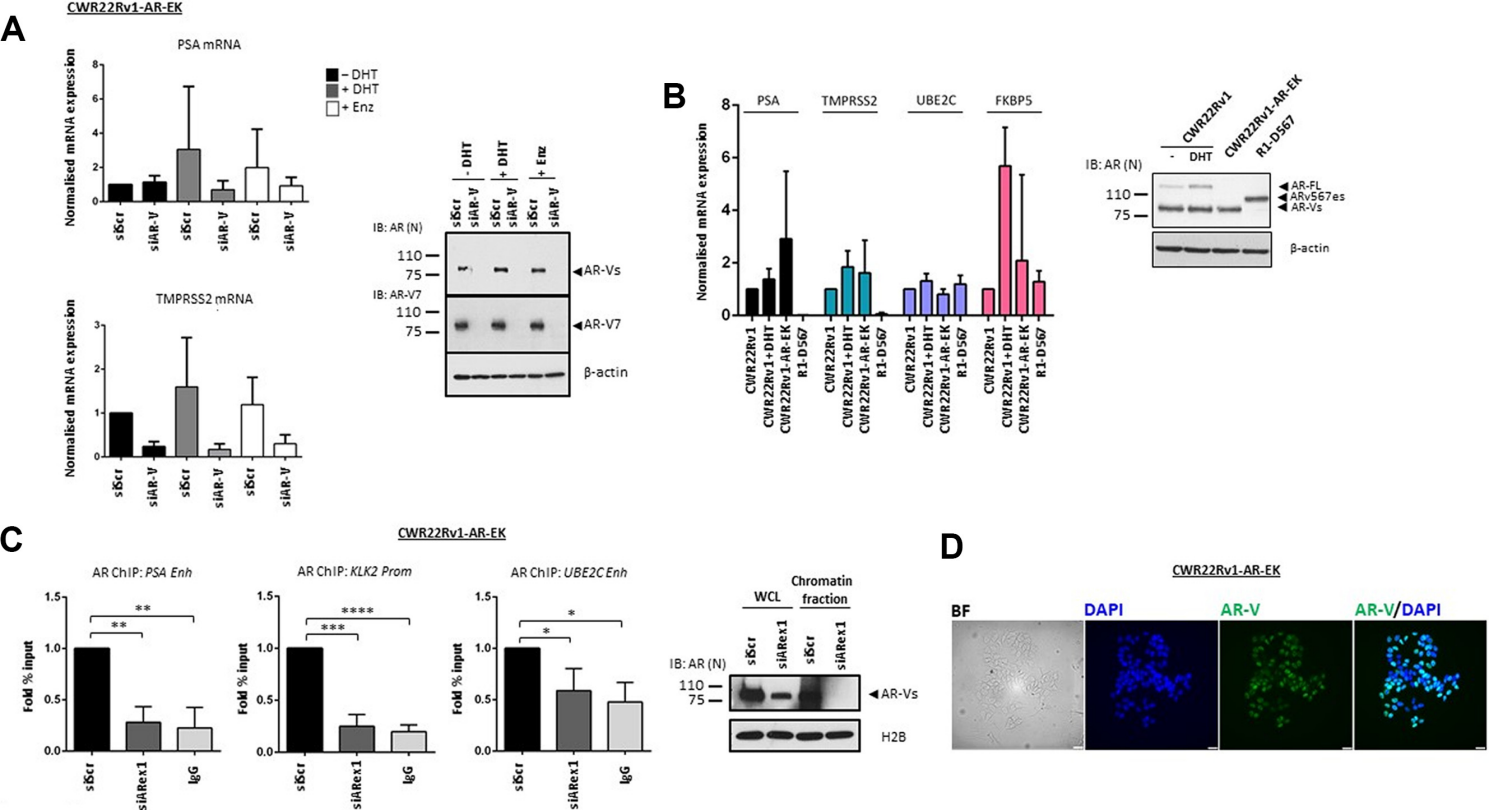
AR-Vs maintain androgenic signalling and bind chromatin in the absence of FL-AR. (**A**) CWR22Rv1-AR-EK cells grown in steroid-depleted media were subject to control (siScr) or AR-V (siAR-V) depletion for 48 hours with either vehicle, 10 nM DHT or 10 μM enzalutamide (Enz) treatment for the final 24 hours before quantitative RT-PCR analysis to assess *PSA* and *TMPRSS2* expression. Data represents the average of three independent experiments ± SD. Validation of AR-V depletion is shown in the accompanying immunoblot (right panel) incorporating N-terminal-binding AR, AR-V7 and β-actin antibodies. (**B**) Comparison of *PSA, TMPRSS2, UBE2C* and *FKBP5* in CWR22Rv1 cells grown in steroid-depleted media supplemented with or without 10 nM DHT, and CWR22Rv1-AR-EK and R1-D567 cells grown in steroid-depleted media by quantitative RT-PCR. Data represents the average of three independent experiments ± SD. Accompanying immunoblot (right panel) of the three cell lines grown in the presence and absence of 10 nM DHT shows AR and β-actin levels. (**C**) CWR22Rv1-AR-EK cells were subject to control (siScr) or AR (siARex1) knockdown for 48 hours before ChIP experiments incorporating either N-terminal AR-binding or control (IgG) antibodies. Data represents the average of three independent experiments ± SD (*, **, ***, **** represent *P*< 0.05, 0.01, 0.001 and 0.0001, respectively as determined using one-way ANOVA). Accompanying immunoblots (right panel) of CWR22Rv1-AR-EK whole cell lysates (WCL) and chromatin fractions, incorporating AR and histone H2B antibodies, demonstrates successful depletion of AR-Vs in siARex1-transfected cells. (**D**) Representative bright field (BF) and immunofluorescence images using an anti-AR antibody (left panel) and DAPI counterstain (right panel) in CWR22Rv1-AR-EK cells. Scale bars are 25 μm.

Whether AR-Vs have the capacity to function as transcriptional regulators independently of FL-AR is debated with evidence suggesting that AR-Vs remain sensitive to the next-generation anti-androgen enzalutamide (37) while other reports, particularly that from the R1-D567 cell line, indicates that AR-Vs support androgenic signalling independently of the full-length receptor (25). To investigate this phenomenon further, CWR22Rv1-AR-EK cells grown in the presence and absence of DHT and enzalutamide were transiently transfected with either scrambled (siScr) or AR-V-targeting (siAR-V) siRNAs and AR-target gene expression was assessed. As shown in Figure 2A (and Supplementary Figure S4), *PSA, TMPRSS2, KLK2, UBE2C* and *ATAD2* remained unchanged in the presence of DHT and enzalutamide, which is consistent with loss of FL-AR, but were all diminished upon depletion of AR-Vs indicating that AR-Vs maintain transactivation of canonical AR-target genes in this cell line. Moreover, expression of AR-target genes in the CWR22Rv1-AR-EK derivative was largely consistent with parental CWR22Rv1 cells grown in both the presence and absence of DHT, and for *UBE2C* and *FKBP5*, was also comparable to the R1-D567 TALEN-modified cell line confirming the ability of AR-Vs to function as transcriptional regulators without FL-AR (Figure 2B). Importantly, AR-target gene expression in CWR22Rv1-AR-EK cells was unaffected by siRNAs targeting the receptor C-terminus (exon 4: siARex4 and exon7: siARex7) further supporting the concept that all androgenic gene expression is driven by AR-Vs in this cell line derivative (Supplementary Figure S5; CWR22Rv1 cells used to demonstrate efficiency of FL-AR depletion by exon 4- and 7-targeting oligonucleotides). Consistently, ChIP using an N-terminal AR antibody demonstrated robust enrichment of AR-Vs at *cis*-regulatory elements of AR-target genes that was down-regulated by exon 1-targeting siRNAs (Figure 2C and Supplementary Figure S6). The presence of AR-Vs in the nucleus was also confirmed by immunofluorescence using an anti-N-terminal AR antibody (Figure 2D).

### AR-V transcriptomics reveals a pro-proliferative role of AR-Vs in CWR22Rv1-AR-EK cells

**Figure 3.**
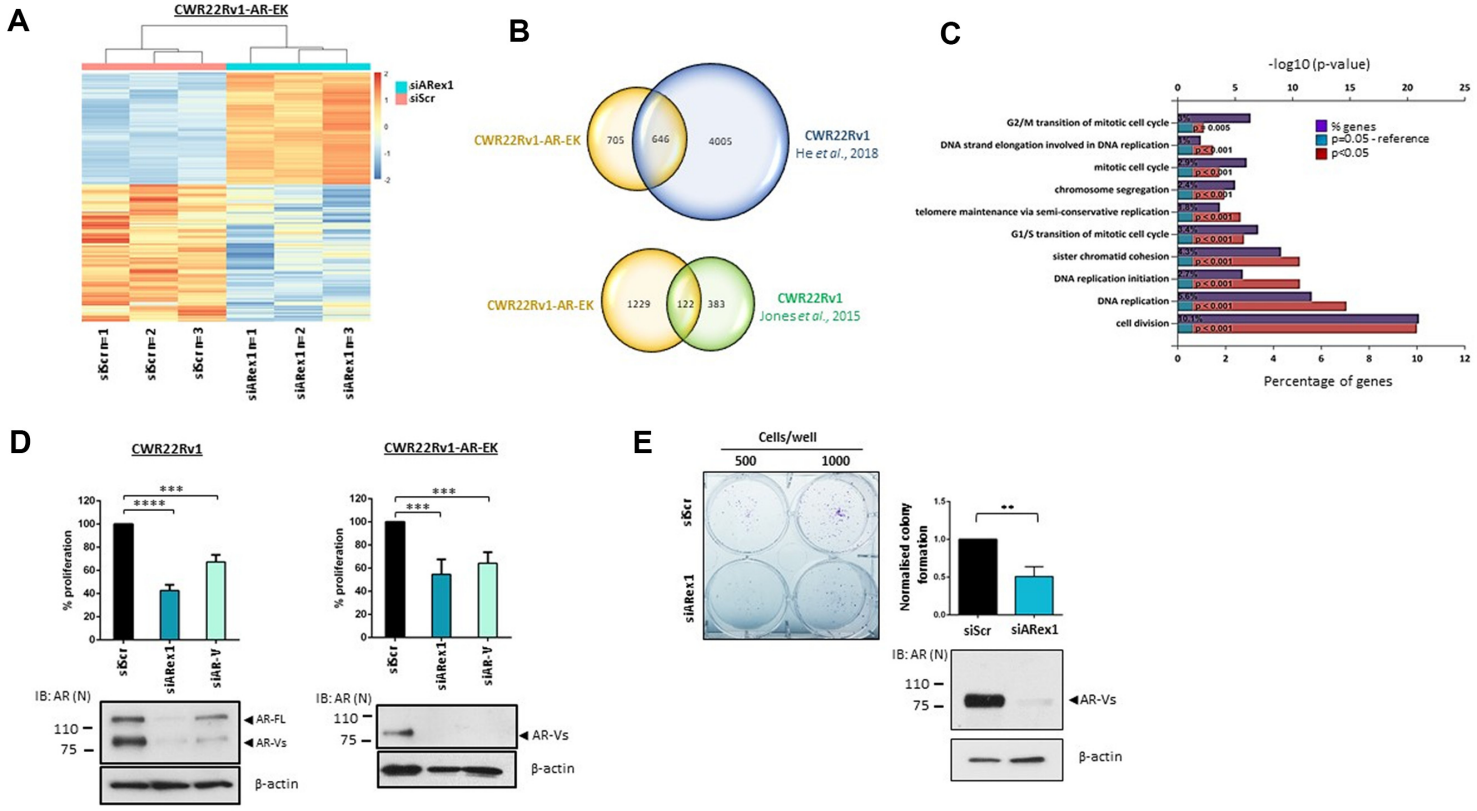
AR-Vs drive proliferative and survival signals in CWR22Rv1-AR-EK cells. (**A**) Heatmap of the log transformed normalised expression of genes up- and down-regulated in triplicate CWR22Rv1-AR-EK cells subject to either control (siScr) or AR-V (siARex1) depletion. The data is row-scaled with red and blue representing relative higher and lower expression, respectively. (**B**) Venn diagrams showing overlap between AR-V-target genes in CWR22Rv1-AR-EK cells and those derived from CWR22Rv1 parental cells depleted of AR-Vs (He *et al.*, 2018 & Jones *et al.*, 2015). (**C**) Functional annotation of AR-V regulated genes in the CWR22Rv1-AR-EK cell line demonstrates that AR-Vs control cell cycle and mitosis-related pathways. The % of genes identified in each pathway are shown alongside statistical significance of genes featuring in these pathways. (**D**) CWR22Rv1 and CWR22Rv1-AR-EK cells were grown in steroid-depleted media and subject to transfection with either control (siScr), FL-AR/AR-V-targeting (siARex1) or AR-V-targeting (siAR-V) siRNAs for 96 hours before analysis of cell proliferation by SRB assays. Data represents the average of three independent experiments ± SD (*** and **** represent *P*< 0.001 and 0.0001, respectively as determined using one-way ANOVA). Lower immunoblotting panels indicate successful depletion of FL-AR and AR-Vs using siARex1 and discriminate knockdown of AR-Vs by siAR-V using an anti-AR antibody. (**E**) Cells transfected as in (D) were subject to clonogenic assays for 2 weeks before quantification. Representative colony numbers are shown in the left panel. Data in the right panel represents the average of three independent experiments ± SD (** represents *P*< 0.01 as determined using a two-tailed Student's *t*-test). Lower panel immunoblot image indicates successful depletion of AR-Vs using an N-terminal AR-binding antibody.

To assess global AR-V transcriptomics, RNA sequencing of CWR22Rv1-AR-EK cells subject to control or AR-V knockdown (Supplementary Figure S7A) was conducted in triplicate. As shown in Figure 3A. (and Supplementary Figure S7B), significantly altered genes in response to AR-V depletion (>1.5 fold cut-off) clustered closely in the three replicates; with 607 and 744 genes demonstrating increased and decreased expression, respectively (Supplementary Tables S1 and S2). Critically, comparison of the CWR22Rv1-AR-EK AR-target gene list with two AR-V-driven gene signatures from CWR22Rv1 cells (Jones *et al.* (22) and He *et al.* (38)) demonstrated respective overlaps of 32% and 48% indicating considerable retention of AR-V function in the genome-edited cell line compared to parental cells (Figure 3B). Moreover, consistent with He *et al.* (38), functional gene annotation (DAVID and Funrich (31)) revealed that pathways involved in cell cycle regulation and mitosis were controlled by AR-V signalling in CWR22Rv1-AR-EK cells (Figure 3C and Supplementary Table S3). This was confirmed by assessing cell proliferation in response to AR-V depletion in both CWR22Rv1 and CWR22Rv1-AR-EK cell lines using two distinct siRNAs; one to deplete FL-AR and all AR-Vs (siARex1) and one to discriminately reduce AR-Vs (siAR-V). As shown in Figure 3D, depletion of all AR isoforms in CWR22Rv1 cells had more robust anti-proliferative effect (∼55%) than knockdown of AR-Vs alone (∼40%) which was to be expected given retention of FL-AR activity in the latter experimental arm. Considering only AR-Vs are expressed in CWR22Rv1-AR-EK cells, both oligonucleotides comparably down-regulated proliferation by ∼40–45%, suggesting that AR-Vs drive a pro-proliferative phenotype (Figure 3D and Supplementary Figure S8). Moreover, clonogenic cell survival assays performed in CWR22Rv1-AR-EK cells demonstrated a 50% reduction in viability upon AR-V depletion substantiating a role for AR-Vs in maintaining cell survival and viability (Figure 3E).

### AR-Vs drive a DNA damage response (DDR) gene signature

**Figure 4.**
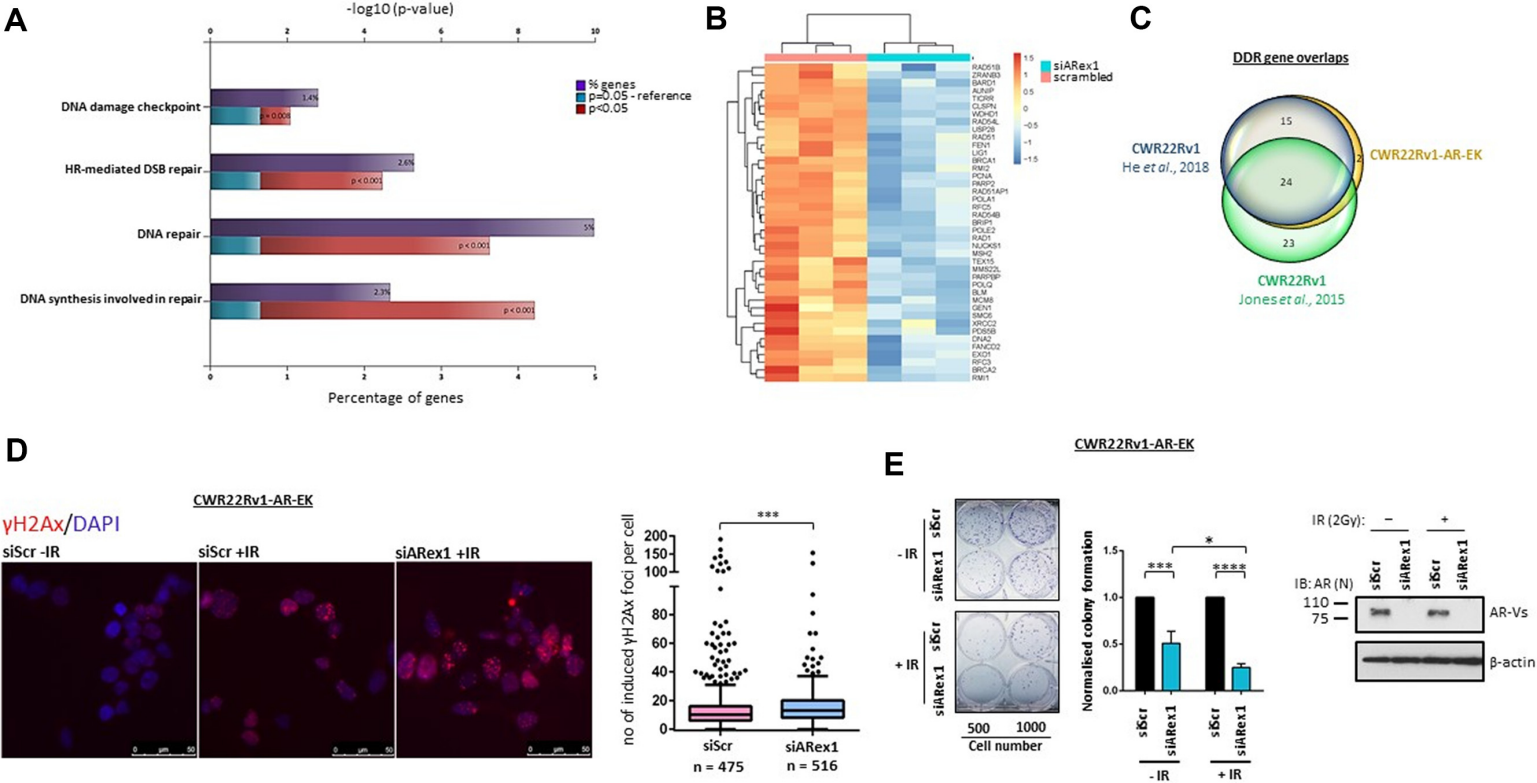
AR-Vs drive a DNA damage response gene signature to desensitise cells to ionising radiation. (**A**) Functional annotation demonstrates that AR-V-regulated genes in the CWR22Rv1-AR-EK cell line control the DNA damage response (DDR). The % of genes identified in each pathway are shown alongside statistical significance of genes featuring in these pathways. (**B**) Heatmap showing log transformed normalised expression of the 41 DDR-associated genes identified in triplicate CWR22Rv1-AR-EK cells transfected with either control (siScr) or AR (siARex1) siRNAs. The data is row-scaled with red and blue representing relative higher and lower expression, respectively. (**C**) Venn diagram demonstrating overlap of the 41 DDR-associated genes identified in CWR22Rv1-AR-EK cells and those identified in CWR22Rv1 cells depleted of AR-Vs (He *et al.*, 2018 and Jones *et al.*, 2015). (**D**) CWR22Rv1-AR-EK cells transfected with control (siScr) or AR-V-targeting (siAR-V) siRNAs were treated with and without 2 Gy ionising radiation and then incubated for 24 hours before quantifying γH2AX foci by immunofluorescence. Representative γH2AX/DAPI images are shown. Scale bars are 50 μm. Data in the right panel represents the average of two independent experiments ± SD (*** represents *P*< 0.001 as determined using a Mann-Whitney test). (**E**) Cells transfected as in (D) were subject to clonogenic assays for 2 weeks with representative colony numbers shown in the left panel. Colonies were quantified in three independent experiments (error bars represent SD and *, ***, **** represent *P*< 0.05, 0.001 and 0.0001, respectively, as calculated using two-way ANOVA).

Several studies have demonstrated that the FL-AR controls expression of genes involved in maintaining DNA integrity (39), including a ‘BRCAness’ signature (26), that potentiates homologous recombination (HR)-mediated DNA repair after ionising radiation-induced DNA damage. Consistent with this phenomenon, treatment of PC with FL-AR antagonists sensitises tumour cells to radiotherapy which is a consequence of compromised AR-driven DDR gene expression and DNA repair (31). However, no studies to date have assessed a direct role of AR-Vs in controlling expression of DNA repair-associated genes and only one report has indicated that AR-V chromatin association and interaction with DNA-PKc is important for DNA repair (40). From a clinical perspective, this is a vital consideration as AR-Vs are refractory to anti-androgens enzalutamide and abiraterone and hence may attenuate PC sensitization to radiotherapy. Functional annotation of our RNA-sequencing data provided evidence that AR-Vs drive a cohort of genes involved in DNA repair (Figure 4A and Supplementary Table S4). Of the 744 down-regulated genes in response to AR-V knockdown in the CWR22Rv1-AR-EK cell derivative, 41 were found to be involved in the DDR (Figure 4B and Supplementary Figure S9); several of which were validated by quantitative RT-PCR, including *RAD54L, PCNA* and *EXO1* (Supplementary Figure S10). In addition to a considerable number of HR-associated genes, AR-Vs regulate base excision and non-homologous end-joining repair pathways (Supplementary Figure S11); many of which demonstrate elevated expression in metastatic PC biopsies compared to localised disease (Grasso *et al.* (32); Supplementary Figure S12). Interestingly, additional *in silico* analysis indicates that the presence of AR-Vs significantly correlates with elevated expression of a number of DDR genes in patient samples, including *EXO1* and *RAD54L* (Supplementary Figure S13; TCGA database) suggesting that AR-Vs may contribute to an elevated DNA repair capability in CRPC. Furthermore, comparison of our AR-V-driven DDR gene signature with both an independent and in-house CWR22Rv1-derived AR-V transcriptome (He *et al.* (26) and Jones *et al.* (22)) indicated respective 95% and 59% overlaps of AR-V-regulated DNA repair genes between the two cell lines, further confirming that loss of FL-AR in the CRISPR-edited cell line has not affected AR-V functionality and, additionally, AR-Vs can maintain a FL-AR-like transcriptome (Figure 4C). This latter assumption was supported by demonstrating robust overlaps between the AR-V DDR gene set (Jones *et al.* (22)) and the FL-AR-driven DDR signature derived from LNCaP cells (31) (Supplementary Figure S14).

To address the phenotypic implications of the AR-V-regulated DDR pathway in response to DNA damage, control or AR-V-depleted CWR22Rv1-AR-EK cells were subject to 2 Gy ionizing radiation (IR) before quantifying γH2AX foci 24 h post-treatment to measure DNA damage repair proficiency. Consistent with compromised expression of the DDR gene signature, cells deficient of AR-Vs (siARexon1) demonstrated elevated γH2AX foci compared to control (siScr) cells indicating AR-V-driven DDR-associated gene expression is important for maintaining DNA integrity in CRPC cells post IR treatment (Figure 4D) and is consistent with a previous report (40). Importantly, ATM activity, as measured by ATM auto-phosphorylation, was found to be unaltered upon AR-V knockdown indicating that failure to successfully repair DNA was not a consequence of attenuated ATM signalling upstream of H2AX phosphorylation (Supplementary Figure S15). Extrapolating this experimental set-up to clonogenic assays, we show that CWR22Rv1-AR-EK cells subject to AR knockdown and IR treatment have significantly reduced survival capacity than either depleting AR-Vs or irradiating cells independently, indicating that failure to drive expression of the DDR gene set, as a consequence of attenuating AR-V signalling, sensitises CRPC to radiotherapy.

### PARP1/2 interacts with AR-Vs and facilitates AR-V activity

**Figure 5.**
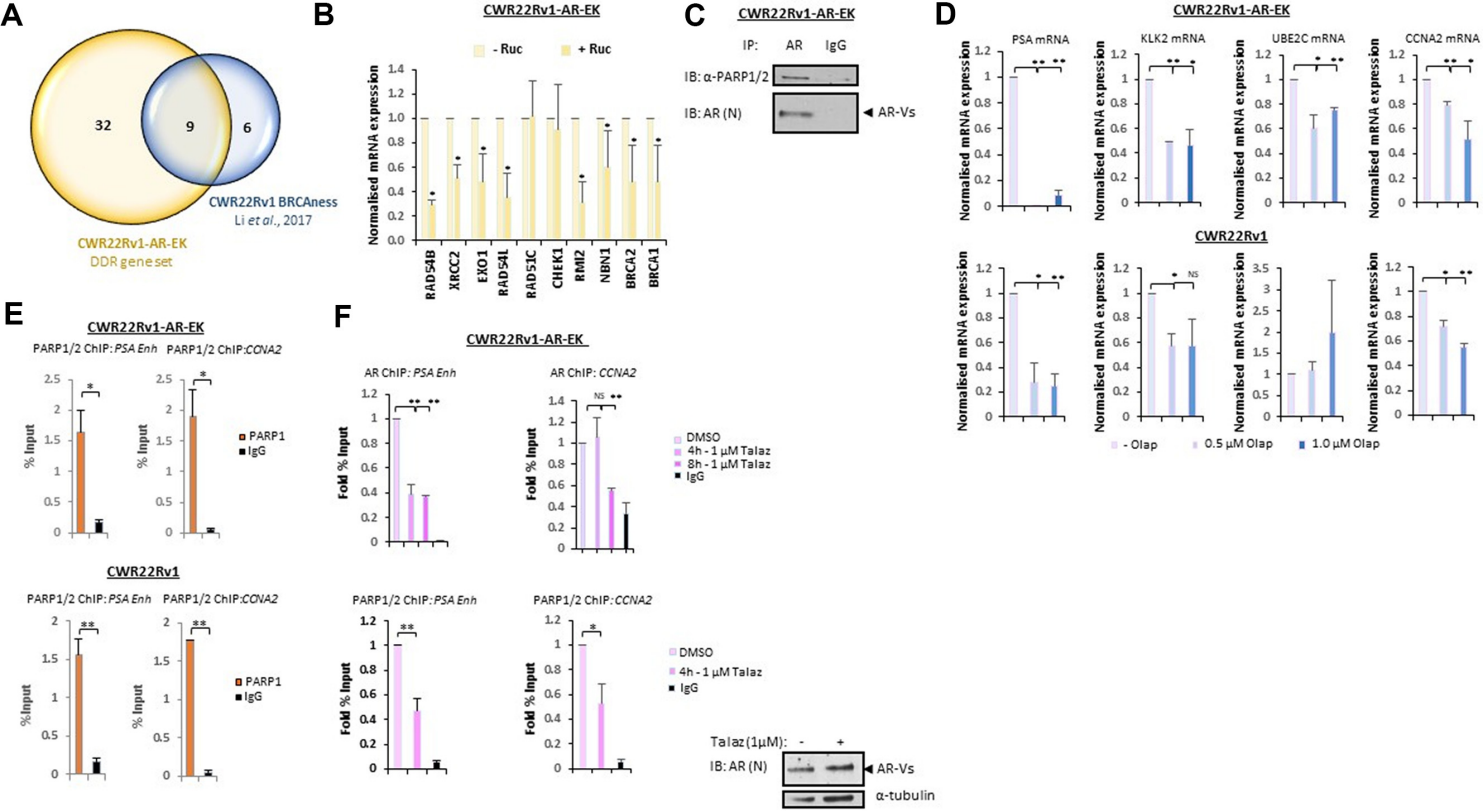
AR-V activity is controlled by PARP1/2. (**A**) Venn diagram indicating overlap between the CWR22Rv1-AR-EK DDR gene set and a ‘BRCAness’ gene signature identified in Li *et al.*, 2017. (**B**) CWR22Rv1-AR-EK cells were treated for 24 h with and without 1 μM rucaparib (Ruc) before qRT-PCR analysis of ‘BRCAness’-associated genes. Data represents two independent experiments ± SD (*P< 0.05 as determined using a two-tailed Student's *t*-test). (**C**) CWR22Rv1-AR-EK cells were subject to immunoprecipitation (IP) using either AR or control (IgG) antibodies and resultant immunoprecipitates were immunoblotted with an anti-PARP1/2 antibody. (**D**) CWR22Rv1-AR-EK and CWR22Rv1 cells were treated with 0.5 and 1 μM olaparib (Olap) for 24 h before quantitative RT-PCR to assess AR-V target gene expression. Data represents the average of three independent experiments ± SD (NS, not significant; *, **P< 0.05 and 0.01, respectively, as determined using a two-tailed Student's *t*-test). (**E**) CWR22Rv1-AR-EK and CWR22Rv1 cells were subject to ChIP using either anti-PARP1/2 or control (IgG) antibodies to assess protein enrichment at AR target genes *PSA* and *CCNA2*. Data represents the average of two independent experiments ± SD (*, ** *P*< 0.05, 0.01, respectively, as determined using a two-tailed student T-test). (**F**) CWR22Rv1-AR-EK cells were treated with and without 1 μM talazoparib (Talaz) for 4 and 8 h before ChIP using AR, PARP and control (IgG) antibodies to assess protein enrichment at AR target genes *PSA* and *CCNA2*. Data represents the average of two independent experiments ± SD (NS, not significant; *, ** *P*< 0.05 and 0.01, respectively, as determined using a two-tailed Student's *t*-test).

Having demonstrated that AR-Vs are important transcriptional regulators of genes involved in DNA repair, we chose to focus on the recently identified ‘BRCAness’ signature which is a FL-AR-regulated gene set important for facilitating HR and sensitivity to PARP inhibitors in the context of PC (26). Attenuating FL-AR signalling with anti-androgens in models of PC down-regulates HR-associated gene expression, compromises the ability for cells to repair double-strand DNA breaks and potentiates elevated sensitivity of cells to PARP blockade (26,41). This phenomenon represents a means of therapeutically inducing synthetic lethality in CRPC. To explore the role of AR-Vs in regulating ‘BRCAness’ genes, quantitative RT-PCR was performed in control and AR-V-depleted CWR22Rv1 cells grown in the presence and absence of enzalutamide. As shown in Supplementary Figure S16, knockdown of AR-Vs in both the presence and absence of anti-androgen significantly downregulated expression of approximately 60% of the ‘BRCAness’ signature, including *BRCA1, BRCA2* and *RAD54B*. Moreover, several ‘BRCAness’ genes were downregulated in CWR22Rv1-AR-EK cells upon AR-V depletion using an N-terminal-targeting siRNA (Figure 5A) and validated using an AR-V-specific siRNA duplex (Supplementary Figure S17) indicating consistency between FL-AR and AR-V signalling in driving HR-mediated DNA repair.

Intriguingly, evidence suggests that in CWR22Rv1 cells, the same ‘BRCAness’-associated genes we have shown to be controlled by AR-Vs are also down-regulated by PARP1 and PARP2 (PARP1/2) inhibitors (26) suggesting that AR-V activity may be regulated by these enzymes. Given that the FL-AR interacts with PARP1 on chromatin, and its transcriptional activity is repressed by inhibitors of PARP1/2 (42), it was important to address if the same mode of regulation applied to AR-Vs. To this end, we firstly assessed the effect of PARP1/2 inhibition on AR-V-driven ‘BRCAness’ genes in the CWR22Rv1-AR-EK cell line. Consistent with the previous report demonstrating attenuated expression of the ‘BRCAness’ signature in response to olaparib-mediated PARP blockade in CWR22Rv1 cells (26), 1 μM of the PARP1/2 inhibitor rucaparib (Ruc) significantly reduced expression of the majority of the ‘BRCAness’ genes in the CRISPR-edited derivative cell line (Figure 5B). We next examined the involvement of PARP activity in the regulation of AR-Vs in CRPC by assessing if the two proteins interacted and whether PARP inhibitors impacted androgenic signalling specifically in the context of AR-Vs. As shown in Figure 5C, AR immunoprecipitates from CWR22Rv1-AR-EK cells immunoblotted with an anti-PARP1/2 antibody demonstrated that AR-Vs interact with PARP1/2. Moreover, by analysing expression of *PSA, KLK2, UBE2C* and *CCNA2* in the presence and absence of 0.5 or 1.0 μM olaparib (Olap), we show that PARP1/2 inactivation reduces AR-V signalling both in 22Rv1-AR-EK cells and, with the exception of *UBE2C*, in the parental cell line grown in steroid-depleted media (Figure 5D). Expanding our analysis to additional PARP inhibitors, both rucaparib and talazoparib (Talaz) down-regulated expression of *PSA, KLK2* and *CCNA2*, but not *UBE2C*, in the two cell lines without effecting AR protein levels (Supplementary Figures S18 and S19) indicating PARP1/2 regulates the transcriptional competency of AR-Vs in a discriminate manner.

ChIP experiments incorporating a PARP1/2 antibody were next performed in parental and CRISPR-edited CWR22Rv1 cell lines to examine if PARP enzymes are recruited to *cis*-regulatory elements of AR target genes. As shown in Figure 5E, PARP1/2 were enriched at enhancer elements of *PSA* and *CCNA2* in both cell lines grown in steroid-depleted media suggesting that the enzymes co-associate with the constitutively chromatin-bound AR-Vs at target loci, as previously demonstrated (22). Moreover, PARP1/2 were detected at the promoter regions of *TMPRSS2* and *FKBP5*, but not at a control region downstream of the *PSA* enhancer (data not shown) suggesting discriminate binding capacities of the enzymes to AR target genes. Consistent with down-regulation of AR-V transcriptional activity, we demonstrated that short-term treatment of the CWR22Rv1-AR-EK and parental derivatives with 1 μM talazoparib significantly reduced AR-V enrichment at a number of androgenic loci, including *PSA, CCNA2* and *KLK2* (Figure 5F, upper panel; Supplementary Figure S20) without impacting total AR-V (and FL-AR) levels in the two cell lines. Talazoparib also significantly reduced the chromatin binding capacity of PARP1/2 at AR target genes (Figure 5E, lower panel; and Supplementary Figure S21) which is consistent with the demonstration that PARP blockade reduces enzyme recruitment to FL-AR binding sites in LNCaP cells (42).

### A feed-forward regulatory loop between AR-Vs and PARP sensitises cells to PARP inhibitors

**Figure 6.**
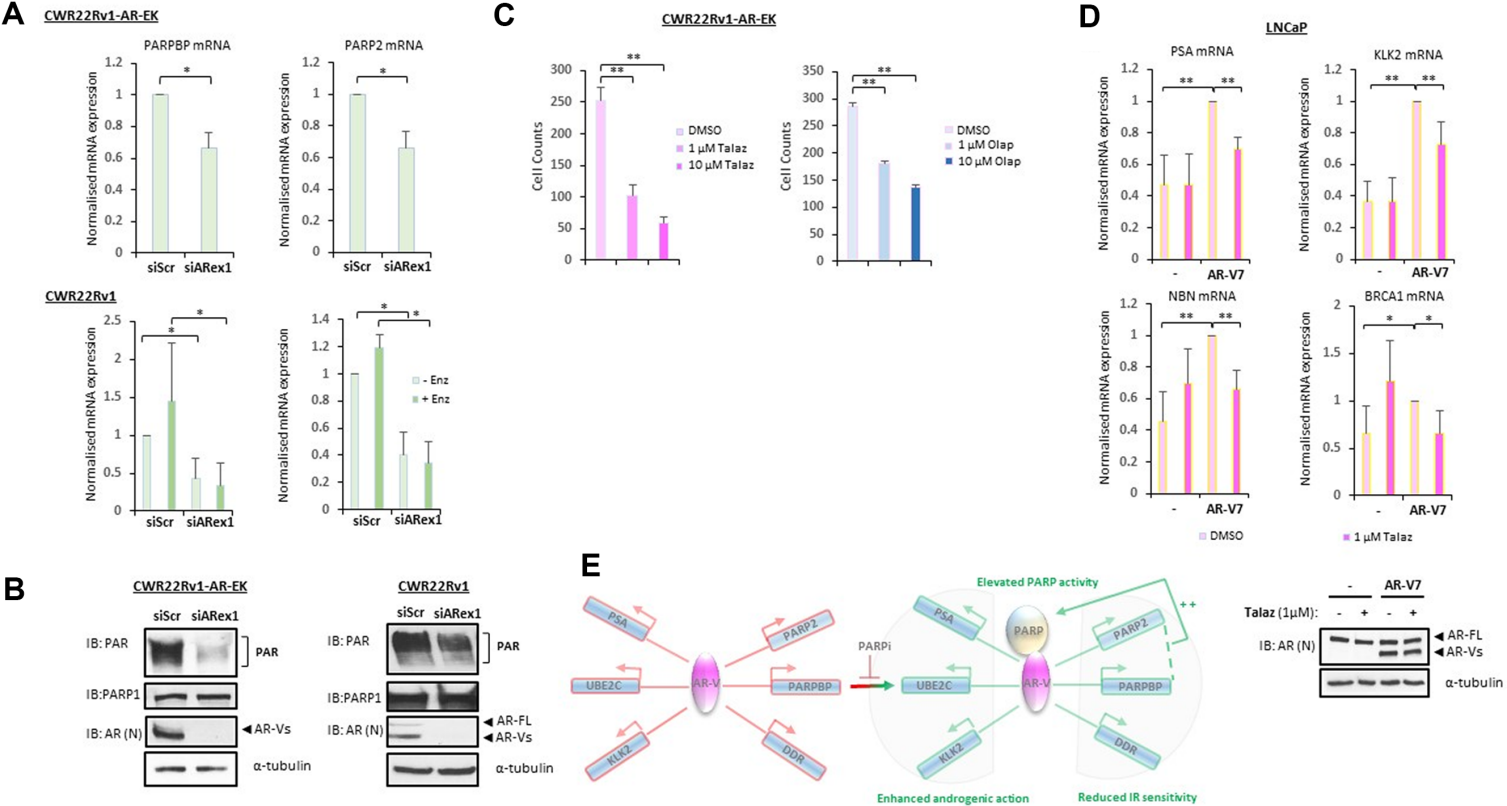
A feed-forward AR-V-PARP regulatory loop facilitates AR-V activity in CRPC. (**A**) PARPBP and PARP2 mRNA levels were analysed by quantitative RT-PCR in CWR22Rv1-AR-EK and CWR22Rv1 cells transfected with control (siScr) or AR (siARex1) siRNAs for 48 h; CWR22Rv1 cells were also grown in the presence and absence of 10 μM enzalutamide. Data represents the average of three independent experiments ± SD (*P< 0.05 as determined using a two-tailed Student's *t*-test). (**B**) Cellular PARP activity was assessed by immunoblotting in CWR22Rv1-AR-EK and CWR22Rv1 cells depleted of AR (siARexon1) using an anti-PAR antibody. Lysates were also probed for PARP1, AR and α-tubulin antibodies. (**C**) CWR22Rv1-AR-EK cells were treated with and without either 1 and 10 μM talazoparib (Talaz) or olaparib (Olap) for 96 hours before cell count analysis. Data represents the average of three independent experiments ± SD (**P< 0.01, as determined using a two-tailed Student's *t*-test). (**D**) LNCaP cells transduced with control or AR-V7-expressing lentivirus for 24 h and then treated with 1 μM talazoparib (Talaz) for an additional 24 h were subject to quantitative RT-PCR to assess expression of AR-target (upper panel) and DDR-associated (lower panel) genes. Data represents the average of three independent experiments ± SD (**P< 0.01 as determined using a two-tailed Student *t*-test). Lower immunoblot image demonstrates ectopic expression of AR-V7 in LNCaP cells transduced with AR-V7-expressing lentivirus. (**E**) Diagrammatic representation of interplay between AR-Vs, the DDR pathway and PARP activity in cells. Expression of AR-V-regulated genes, including those involved in the DDR, is enhanced by PARP1/2. The ability of AR-Vs to up-regulate PARPBP and PARP2 expression, which enhance cellular PARP activity, potentiates the existence of a feed-forward regulatory loop in CRPC.

Interrogation of our RNA sequencing data from the CWR22Rv1-AR-EK cell line indicated that expression of *PARP2* and the PARP1-binding protein *PARPBP* were down-regulated upon depletion of AR-Vs (Supplementary Figure S9). This was also evident in CWR22Rv1 cells grown in steroid-depleted conditions and subject to AR-V knockdown (22) suggesting that AR-Vs control expression of key PARP enzymes and regulators in CRPC cells. To validate our findings, quantitative RT-PCR was conducted in the parental and CRISPR-edited CWR22Rv1 cell lines depleted of AR-Vs. As shown in Figure 6A, while PARPBP and PARP2 mRNA levels were unaffected by enzalutamide treatment, they were significantly down-regulated upon knockdown of AR in the two cell lines suggesting that AR-Vs are able to sustain expression of these two genes in the absence of FL-AR activity. Given that PARPBP enhances PARP1 activity (43) and PARP2 contributes to cellular PARP activity, we postulated that global PARP function would be compromised in cells depleted of AR-Vs. Immunoblotting of AR-depleted CWR22Rv1-AR-EK and CWR22Rv1 cell lysates with a PAR antibody, which is a surrogate marker for cellular PARP activity, demonstrated that PARP enzymatic function was reduced in cells subject to AR knockdown (Figure 6B) indicating that AR-Vs are able to elevate PARP catalytic capacity in CRPC. Importantly, PARP1 levels were unaffected by AR depletion suggesting that loss of PARP2 and PARPBP is sufficient to attenuate enzymatic function in cells. Based on the demonstration that AR-V transcriptional competency is enhanced by PARP1/2, including the target genes *PARP2* and *PARPBP*, our data provides evidence of a feed-forward regulatory loop that enables persistence of AR-V signalling to potentiate growth of CRPC cells. Critically, previous reports have indicated that growth of the CRPC cell lines LNCaP, VCaP and CWR22Rv1 is diminished in response to PARP inhibitor treatment (35) and therefore it was important to address if CWR22Rv1-AR-EK cells remain sensitive to PARP blockade. Using a cell proliferation read-out, we show that both talazoparib and olaparib significantly reduced growth of the CRISPR-edited derivative (Figure 6C) suggesting that in AR-V positive CRPC, PARP1/2 blockade may represent a novel strategy to repress receptor splice variant function and, in-turn, attenuate growth of advanced disease.

To provide additional confirmation that PARP activity is required for AR-V activity, LNCaP cells transduced with control or AR-V7-expressing lentivirus, and treated with 1 μM talazoparib, were subject to quantitative RT-PCR to assess the effect of AR-V7 expression and PARP inhibition on AR-V target gene expression. As shown in Figure 6D, as well as *PSA* and *KLK2*, ectopically-expressed AR-V7 enhanced expression of the HR-associated genes *NBN* and *BRCA1* (and *RAD21, CHEK1, XRCC2, BMC1, EXO1*, Supplementary Figure S22) which confirms that AR-Vs can upregulate genes involved in DNA repair. Importantly, AR-V7-mediated activation of several genes, including *PSA, KLK2, NBN, BRCA1, RAD21* and *CHEK1*, were inactivated by PARP inhibition, further confirming that PARP controls AR-V function in CRPC. That not all genes were impacted by PARP blockade supports the concept that, like many identified AR co-regulators, PARP activity is required for a discriminate number of AR-V target genes controlling androgenic and DNA repair signalling pathways (Figure 6E). In summary, our novel CRISPR knock-in cell line has provided unequivocal evidence that AR-Vs are important for CRPC growth, maintaining DNA integrity and are controlled, in part, by a feed-forward regulatory loop involving PARP enzymes which provides new avenues for therapeutically targeting AR-V positive CRPC.

## DISCUSSION

Although most PC patients respond favourably to initial hormone therapy all eventually relapse to more aggressive CRPC (4,5). At this stage, treatments are limited, but next-generation anti-androgens, such as enzalutamide and apalutamide, show efficacy in ∼50% of patients (14,15). Unfortunately, expression of alternatively spliced forms of AR, termed AR-Vs, in a large cohort of CRPC patients drives disease progression unchallenged by next-generation treatments (13,16). It is critical, therefore, that there is considerable focus on improving our understanding of how AR-Vs function in disease to identify tractable targets for effective therapies in AR-V-expressing CRPC.

Critically, the paucity of good models to assess specific co-regulator dependencies and the transcriptome of AR-Vs has made it challenging to define the precise function of receptor splice variants in CRPC. This is, in part, a consequence of not being able to fully distinguish between the activities of AR-Vs and FL-AR which are co-expressed in cell lines such as VCaP and CWR22Rv1. The development of the TALEN-engineered R1-D567 cell line which expresses the single receptor splice variant AR-v567es has been a valuable addition to the PC model toolbox; providing an important insight into co-regulator requirements of AR-Vs, such as dependency on the BET family of bromodomain-containing proteins (25,44). There remains, however, a need to have additional cell lines that recapitulate the clinical scenario. Given that VCaP and CWR22Rv1 cells express multiple AR-Vs, a phenomenon observed in circulating tumour cells (18), it is important that new models express several clinically-relevant AR-Vs to mimic CRPC. Co-expression of AR-Vs in the same patient may indicate that AR splice variants form a more complex interaction network with one another to what was initially thought and they may mediate signalling in certain combinations. Two recent studies by Chen *et al.* (45) and *Cai et al*. (46) highlighted specific chromatin interactions between AR-V7 and the Hox13B and ZFN co-regulators, respectively, and provided transcriptomic analyses in parental CWR22Rv1 cells depleted of AR-V7. In contrast to these studies which exclusively focus on AR-V7 and acknowledging the presence of more than one AR-Vs in clinical samples, we intended to profile all AR-Vs and look at the pathways they regulate in concert. To address this, we have developed the first of its kind CRISPR-derived CRPC cell line derivative, modelled in CWR22Rv1 cells, that has lost FL-AR expression, but retains expression of all endogenous AR-Vs. This new cell line, termed CWR22Rv1-AR-EK (AR-exon knockout) has an edited *AR* gene containing a knock-in stop codon to prevent synthesis of FL-AR protein, and wild-type exons encoding the N-terminal transactivation and DNA-binding domains, to enable expression of all AR-Vs nascent to the parental cell line. Key validation experiments have indicated that FL-AR is not detectable in this new derivative using a host of anti-AR antibodies in IP and immunoblotting experiments, while also demonstrating no off-target CRISPR activity at several of the highest-ranked off-target loci. One potential issue with the use of CWR22Rv1 cells is the presence of an intragenic duplication within the *AR* gene, encompassing exon 3, that could impact on synthesis or activity of AR isoforms. Indeed, a TALEN-engineered CWR22Rv1 cell derivative lacking this duplication (termed 22Rv1-undup1–3) demonstrated elevated FL-AR levels and reduced expression of AR-Vs suggesting that generation of AR isoforms may be distinctly regulated in the CWR22Rv1 model containing the aberrant *AR* gene (47). Importantly, however, the sensitivity of AR-V expression to anti-androgens and transactivation capacity of AR-Vs in the 22Rv1-undup1–3 cell line was consistent with other AR-V-expressing models suggesting that the altered AR gene locus in parental CWR22Rv1 cells may not have a marked impact on AR-V functionality. Therefore, we believe this cell line offers a genuinely novel model to unequivocally assess the combined function of a number of clinically-relevant AR-Vs without interference from FL-AR which could expedite discriminate AR-V-targeting drug development campaigns.

As expected, analysis of AR-target gene expression in the CWR22Rv1-AR-EK cell line demonstrated consistency with parental CWR22Rv1 cells in that *PSA, KLK2, UBE2C* and *CCNA2* remain refractory to DHT and anti-androgen treatment. Importantly, knockdown of all AR-Vs in CWR22Rv1-AR-EK cells using either exon 1- or cryptic exon 3-targeting siRNAs markedly down-regulated chromatin enrichment of AR-Vs at *cis*-regulatory elements and attenuated expression of these genes suggesting that receptor splice variants are necessary and sufficient for driving an androgenic gene signature and function unhindered in the absence of FL-AR. Moreover, these findings suggest that transactivation of canonical AR-target genes is driven by AR-V homodimers and is consistent with observations in PC3 cells ectopically expressing AR-V7 and AR^V567es^ which demonstrated both homo- and heterodimers between the distinct AR-V isoforms (48).

RNA sequencing was next conducted to determine global transcriptomics of AR-Vs in the CRISPR-derived cell line. Consistent with a recent report indicating that AR-V7 both positively and negatively regulates target genes (49), depletion of AR-Vs in the CWR22Rv1-AR-EK cells resulted in a total of 1351 differentially-expressed genes, with 607 up-regulated and 744 down-regulated in response to loss of AR-V signalling. Comparing our RNA sequencing data to two previous AR-V transcriptomics studies undertaken in parental CWR22Rv1 cells (Jones *et al.* (22) and He *et al.* (38)), which identified 506 and 4651 differentially-expressed genes, respectively, we demonstrate considerable overlaps in AR-V-target gene signatures of 32% and 48%, respectively, suggesting that a conserved core of AR-V-regulated genes exists. However, the different experimental approaches utilized to assess AR-V activity between the studies, particularly with respect to how FL-AR was inactivated, the extent of AR-V knockdown and sequencing versus micro-array platforms, is likely to contribute to a considerable degree of variation in the overall numbers of differentially expressed genes reported. This is particularly apparent when comparing AR-V repressed genes between our data-set and the Cato *et al.* (49) study in which the latter was performed in the LNCaP95 model cell line. Importantly, functional annotation of the CWR22Rv1-AR-EK AR-V-driven gene signature provided evidence for a role of AR-Vs in cell cycle and mitotic pathways which is consistent with both Jones *et al.* (22) and He *et al.* (38) suggesting that the core overlapping genes from the distinct AR-V transcriptomes play key roles in regulating cell fate. In keeping with this observation, both proliferation and clonogenics assays validated AR-Vs as key regulators of cell growth and viability in CWR22Rv1-AR-EK cells.

Outside of cell cycle regulation, one of the other highly ranked AR-V-regulated pathways identified by functional clustering was DNA repair. This was an exciting observation given the number of recent reports describing a role for FL-AR as a regulator of the DDR (26,39,41). We identified 41 AR-V-regulated genes involved in distinct aspects of the DDR, including HR and non-homologous end joining (NHEJ), which showed considerable overlap with other AR-V transcriptomes (22,38), providing evidence that, like FL-AR, AR-Vs up-regulate a DDR signature to maintain DNA integrity. Consistent with this, reduced expression of these genes by depleting AR-Vs prevented resolution of IR-induced DNA breaks, as measured by γH2AX foci, and sensitised cells to ionising radiation. This finding is in-line with recent evidence from the R1-D567 cell line describing AR-Vs as regulators of cellular DNA repair proficiency (40). Of note, however, is that while radiation treatment induced DDR gene expression in TALEN-engineered R1-D567 cells, it was unlikely to be driven by the AR-v567es variant suggesting that key differences in controlling the DDR exist in these two exclusively AR-V-expressing cell lines. We speculate that the multiple AR-Vs present in the CWR22Rv1-AR-EK cell line have a greater capacity to drive and sustain key pathways in CRPC, akin to the FL-AR, compared to the single receptor splice variant AR-v567es. Similarly, neither Chen *et al.* (45) nor Cai *et al.* (50) indicated any involvement of AR-V7 in DDR regulation in CWR22Rv1 cells. This might suggest that AR-V7 depletion is compensated by other AR-Vs expressed in this cell line and shows that a single receptor splice variant might not be sufficient to drive DDR individually but it may require the activity of other AR-Vs to ultimately mediate DDR signalling in concert.

From a translational standpoint, the ability to sensitise PC to IR by inactivating the FL-AR-driven DDR pathway with anti-androgens has improved efficacy of radiotherapy (RT) in the clinic. Our data and that of others (40), however, would suggest that in AR-V-expressing CRPC cells, castration-induced IR sensitization would be ineffective as the DDR would be maintained by AR-Vs. Whether this is important in the clinical setting, where RT is combined with castration modalities to treat locally-confined PC remains to be seen. Importantly, in addition to the observed interplay between AR signalling, the DDR and sensitivity to radiation, the identification of a ‘BRCAness’ gene signature that is up-regulated by the FL-AR to elevate cellular HR competency has provided new therapeutic avenues based on the concept of synthetic lethality between androgen signalling and PARP inhibitors (26). By blocking this FL-AR-induced ‘BRCAness’ gene set with enzalutamide in the LNCaP and VCaP cell lines, cells were sensitized to the PARP inhibitor olaparib; providing evidence of a novel combined treatment strategy involving anti-androgens and PARP blockade to potentiate enhanced tumour cell killing. The fact that we have identified AR-Vs as drivers of a considerable number of ‘BRCAness’-associated genes in both CWR22Rv1-AR-EK and parental CWR22Rv1 suggests that a similar synthetic lethality relationship may be exploitable in AR-V positive PC. Although potentially feasible using CRISPR and siRNA-mediated depletion strategies in cell line models, the failure to currently inactivate AR-Vs using clinically-relevant agents remains a critical drawback to test and validate this concept in patients.

One crucial piece of evidence recently published demonstrated that olaparib treatment down-regulated the ‘BRCAness’ gene signature in CWR22Rv1 cells (26). We mimicked this finding by depleting AR-Vs, suggested that PARP may be directly involved in regulating the transcriptional activity of AR-Vs. The fact that PARP1/2 controls transcriptional competency of the FL-AR (42) suggests that such a mode of AR-V regulation is feasible. Using several PARP1/2 inhibitors, we provide evidence that AR-V transcriptional activity is regulated, in part, by PARP enzymes and both androgenic and DDR target genes show attenuated expression upon enzyme blockade. Mechanistically, PARP1/2 associates with *cis*-regulatory elements of AR-V-target genes in parental and CRISPR-modified CWR22Rv1 cells and PARP inactivation down-regulates both AR-V and PARP1/2 chromatin-binding. Further interrogation of our transcriptomics data derived from CWR22Rv1 and CWR22Rv1-AR-EK cells identified that both *PARP2* and *PARBP* were down-regulated in response to AR-V knockdown suggesting the existence of a feed-forward regulatory loop between PARP1/2 and AR-Vs that can help elevate and sustain the expression of AR-V-mediated androgenic and DDR gene signatures. Consistent with these findings, cellular PARP activity was shown to be diminished in cells depleted of AR-Vs, suggesting that down-regulated expression of both *PARP2* and *PARPBP* contributes to compromised cellular enzymatic activity. Intriguingly, this finding is at odds with recent data demonstrating that anti-androgen treatment up-regulates PARP activity in FL-AR-expressing C4–2 cells which was suggested to be a redundancy mechanism driven by loss of AR-mediated HR-associated gene expression (41). One explanation for this discrepancy could be that depletion of AR-Vs in CWR22Rv1 cells over prolonged periods stalls cells in the G1 phase of the cell cycle (22) which is associated with diminished PARP function (51). Hence, our findings that PAR levels are depleted in AR-V knockdown CWR22Rv1-AR-EK cells may, in part, be a consequence of cell cycle stalling in the G1 phase of the cell cycle in addition to the direct regulation of AR-Vs on *PARP2* and *PARPBP* expression.

Overall, the development of this novel CRISPR-mediated knock-in cell line has provided an unequivocal read-out for AR-V transcriptomics and highlighted new modes of AR-V regulation that suggest new pharmacological sensitivities in advanced PC patients who express receptor splice variants. By down-regulating AR signalling and expression of a DDR gene signature, PARP inhibitors may concurrently attenuate androgenic cell growth and promote ‘BRCAness’ to sensitise cells to DNA damaging agents.

## DATA AVAILABILITY

RNA-seq data has been deposited into NCBI Gene Expression Omnibus (GEO) (accession number: GSE126306).

## Supplementary Material

gkz286_Supplemental_File
