# Supplementary material for: A novel CRISPR-engineered prostate cancer cell line defines the AR-V transcriptome and identifies PARP inhibitor sensitivities
Source: Nucleic Acids Res. 2019 Apr 22;47(11):5634–47. doi: 10.1093/nar/gkz286 (PMC6582326; doi:10.1093/nar/gkz286)
Supplement: gkz286_Supplemental_File [file gkz286_supplemental_file.pdf]

Supplementary Information to accompany Kounatidou et al.

Contents

Figure legends (S1-S21) **Pages 2-8**

Tables (S1-S6) **Pages 9-18**

Supplementary Figures (S1-S22) **Pages 19-41**

Figure legends

Supplementary Figure S1. Validating CRISPR knock-in strategy in CWR22Rv1 cells. a. CWR22Rv1 cells were transiently transfected with either Cas9/gRNA_1- or Cas9/gRNA_2-encoding plasmids and immunofluorescence images were taken to demonstrate successful expression of the plasmid-derived GFP marker (left panel). Resultant SURVEYOR endonuclease assay of CRISPR target amplicon of the *AR* gene exon 5 locus from Cas9/gRNA_1- and Cas9/gRNA_2-transfected CWR22Rv1 cells (* indicates successful cleavage of hetero-duplexes generated as a consequence of mixing equal amounts wild-type and CRISPR-modified amplicons). **b.** TIDE analysis of resultant sequencing data derived from Cas9/gRNA_1- and Cas9/gRNA_2-trasfected cells (PAM sites for both CRISPR complexes are shown at top of right panel). **c.** Diagrammatic representation of donor template used to knock-in stop codon (TAA) into exon 5 of the AR gene. TTAA sequence represents an *Mse* I restriction enzyme site to enable restriction fragment length polymorphism (RFLP) analysis. F- and R-labelled arrows indicate primers used to amplify DNA from clonal cell populations to identify incorporation of donor template into the host genome. **d.** Successful detection of donor template incorporation into CWR22Rv1 cells as indicated by the generation of two DNA fragments by *Mse* I-mediated RFLP to form the derivative cell line CWR22Rv1-AR-EK.

Supplementary Figure S2. Validating loss of FL-AR in CWR22Rv1-AR-EK cells. CWR22Rv1- and CWR22Rv1-AR-EK-derived lysates were subject to immunoblotting using four distinct AR N-terminal-targeting antibodies: AR N20 (Santa Cruz Biotechnology - discontinued), AR-BD (BD Pharmingen), AR ab74272 (Abcam) and AR-441 (Santa Cruz Biotechnology). Short and long exposure were performed to enable detection of FL-AR in CWR22Rv1 parental cells. α-tubulin was used as loading control.

Supplementary Figure S3. Validating genomic integrity of CWR22Rv1-AR-EK cells. a. Bright field images of CWR22Rv1 and CWR22Rv1-AR-EK cells were taken at a 10x magnification to demonstrate similar morphology of the two cell lines. **b.** CWR22Rv1 and CWR22Rv1-AR-EK share equivalent genomic SNP markers as determined using conventional cell line authentication. **c.** Predicted potential CRISPR off-target sites were amplified using specific primers and resultant amplicons were analysed by TIDE. Sequencing chromatograms of the three highest ranked off-target loci within *COL1A2*, *KMT2B* and *SLC7A8* genes are shown for parental and CWR22Rv1-AR-EK cells (left panel) and analysed by TIDE (right panel).

Supplementary Figure S4. AR-V depletion in CWR22Rv1-AR-EK down-regulates androgenic gene expression. CWR22Rv1-AR-EK cells grown in steroid-depleted media were subject to control (siScr) or AR-V (siAR-V) depletion for 48 hours with either vehicle, 10 nM DHT or 10 μM enzalutamide (Enz) treatment for the final 24 hours before quantitative RT-PCR to assess *UBE2C, ATAD2* and *KLK2* expression. Data represents the average of three independent experiments +/- SD.

Supplementary Figure S5. AR target gene expression in CWR22Rv1-AR-EK is driven exclusively by AR-Vs. a. CWR22Rv1-AR-EK cells grown in steroid-depleted media were subject to transfection with control (siScr) or FL-AR-targeting siRNAs (siARex4 and siARex7) for 48 hours with either vehicle or 10 nM DHT treatment for the final 24 hours before quantitative RT-PCR to assess *PSA*, *KLK2* and *UBE2C* expression. Data represents the average of three independent experiments +/- SD. **b.** Cell lysates harvested from the parallel experiment were subject to western blot analysis using anti-AR and α-tubulin antibodies to demonstrate that unlike siARex1, both siARex4 and siARex7 have no impact on AR-V levels in CWR22Rv1-AR-EK cells (left panel). Using CWR22Rv1 parental cells, siARex4 and siARex7 were able to successfully deplete FL-AR levels (right panel).

Supplementary Figure S6. AR-Vs associate with AR-target genes in CWR22Rv1-AR-EK cells. CWR22Rv1-AR-EK cells were subject to control (siScr) or AR (siARex1) knockdown for 48 hours before ChIP experiments, incorporating either N-terminal AR-binding or control (IgG) antibodies. Data represents the average of three independent experiments +/- SD (** represents $p<0.01$ as determined using one-way ANOVA).

Supplementary Figure S7. Validating samples for RNA sequencing analysis. a. Triplicate CWR22Rv1-AR-EK samples transfected with either control (siScr) or AR-V-targeting (siARex1) siRNAs were subject to quantitative RT-PCR and immunoblotting to assess effect on AR-target gene expression (left panel) and validate AR-V depletion (right panel). Data represents the average of three independent experiments +/- SD (** represents $p<0.01$ as determined using a two-tailed student T-test). **b.** MA plot of RNA sequencing data from three experimental replicates showing statistically significant gene expression changes ($p<0.01$) above and below the 0 y-intercept representing up- and down-regulated genes, respectively, shown in red.

Supplementary Figure S8. Reduced CWR22Rv1-AR-EK cell growth in response to AR-V depletion. CWR22Rv1-AR-EK and CWR22Rv1 cells grown in steroid-depleted media were subject to control (siScr), total AR (siARex1) or AR-V only (siAR-V) knockdown for 96 hours before 10 x magnification bright field images were taken.

Supplementary Figure S9. CWR22Rv1-AR-EK RNA sequencing demonstrates down-regulation of DDR-associated genes in response to AR-V depletion. Expression of the 41 DDR-associated genes

identified to be consistently and significantly down-regulated (* $p<0.01$) in response to AR-V knockdown.

Supplementary Figure S10. Validation of DDR-associated gene regulation by AR-Vs. a. CWR22Rv1-AR-EK cells depleted of AR-Vs (siARex1) for 48 hours were subject to quantitative RT-PCR to assess expression of several DDR-associated genes. Data represents the average of three independent experiments +/- SD (* $p< 0.05$ as determined using a two-tailed student T-test).

Supplementary Figure S11. Defining individual DNA repair pathways of the AR-V regulated DDR-associated genes. The 41 DDR-associated genes demonstrated to be regulated by AR-Vs in the CWR22Rv1-AR-EK cell line were sub-compartmentalised into their associated DNA repair pathway using FunRich.

Supplementary Figure S12. Expression AR-V-regulated DDR-associated genes is elevated in CRPC. Expression of several AR-V-regulated DDR-associated genes from the CWR22Rv1-AR-EK cell line was assessed in benign hyperplasia (BPH), localised PC and CRPC samples *in silico* using the Grasso *et al*., (2012) data set (ns is non-significant; *, **, ***, **** represent $p< 0.05$, 0.01, 0.001 and 0.0001, respectively).

Supplementary Figure S13. The presence of AR-V7 in patient samples correlates with elevated expression of several DDR-associated genes. a. Expression of the 41 AR-V-regulated DDR-associated genes identified in CWR22Rv1-AR-EK cells were assessed in the TCGA dataset using cBioPortal with the presence of AR-V7 applied as an additional clinical parameter to enable quantification of expression in samples positive and negative for the receptor splice variant. **b.** Of the 41 genes, 9

demonstrated significantly elevated expression in PC samples that co-expressed AR-V7 (n=84) as calculated using a Bioconductor TCGAbiolinks package (version 2.9.0).

Supplementary Figure S14. Overlapping DDR-associated genes between AR-Vs and FL-AR. Venn diagram demonstrating overlapping DDR-associated gene signatures driven by AR-Vs in CWR22Rv1 cells (Jones *et al*., 2015) and FL-AR in LNCaP cells (Polkinghorn *et al*., 2012).

Supplementary Figure S15. Examining ATM activation status in CWR22Rv1-AR-EK cells depleted of AR-Vs. CWR22Rv1-AR-EK cells were transfected with control (siScr) or AR-targeting (siARex1) siRNAs for 24 hours before 2 Gy irradiation treatment and incubation for an additional 24 hours before immunblotting using total ATM, phospho-ATM, AR and β-actin antibodies.

Supplementary Figure S16. AR-V depletion in CWR22Rv1 cells reduces expression of 'BRCAness' genes. CWR22Rv1 cells grown in steroid-depleted medium were transfected for 24 hours with control (siScr) or AR-targeting siRNAs (siARex1) before 24 hour treatment with and without 10 µM enzalutamide (Enz) were subject to quantitative RT-PCR to assess 'BRCAness' gene expression. Data is presented as a heatmap (upper panel) and in graphical form; both represent the mean +/- SD of three independent experiments (* $p<0.05$ as determined using a two-tailed student T-test).

Supplementary Figure S17. AR-V depletion in CWR22Rv1-AR-EK cells reduces expression of 'BRCAness' genes. CWR22Rv1-AR-EK cells grown in steroid-depleted medium were transfected with control (siScr) or AR-V-targeting siRNAs (siAR-V) for 48 hours and were subject to quantitative RT-PCR to assess gene expression. Data represent the mean +/- SD of three independent experiments

(*p<0.05 as determined using a two-tailed student T-test). Parallel samples were subject to immunoblotting using AR and α-tubulin antibodies.

Supplementary Figure S18. PARP inhibition using talazoparib reduces AR-V activity. CWR22Rv1-AR-EK cells were treated with 1 μM talazoparib (Talaz) for 24 hours before quantitative RT-PCR to assess AR-V target gene expression. Data represents three independent experiments +/- SD (***p<0.001 as determined using a two-tailed student T-test). Parallel samples were subject to immunoblotting using AR and α-tubulin antibodies.

Supplementary Figure S19. PARP inhibition using rucaparib reduces AR-V activity. CWR22Rv1-AR-EK and CWR22Rv1 cells were treated with 0.5 and 1 μM olaparib (Olap) for 24 hours before quantitative RT-PCR to assess AR-V target gene expression. Data represents three independent experiments +/- SD (***p<0.001 as determined using a two-tailed student T-test).

Supplementary Figure S20. PARP inhibition reduces AR enrichment at target genes. CWR22Rv1-AR-EK (**a.**) or CWR22Rv1 cells (**b.**) treated for 4 (and 8) hours with 1 μM talazoparib (Talaz) were subject to ChIP using either anti-AR or control (IgG) antibodies to assess AR enrichment at AR target gene promoters *PSA* and *KLK2*. Data represents two independent experiments +/- SD (*, ** p<0.05, 0.01, respectively, as determined using a two-tailed student T-test). Immunoblotting of resultant CWR22Rv1 cell lysates using AR and α-tubulin antibodies is shown in the right panel.

Supplementary Figure S21. PARP inhibition reduces AR enrichment at target genes. CWR22Rv1-AR-EK cells were treated with and without 1 μM talazoparib (Talaz) for 4 hours before ChIP using PARP1/2 and control (IgG) antibodies to assess protein enrichment at AR target gene promoters *PSA* and *KLK2,*

and the *TMPRSS2* enhancer. Data represents two independent experiments +/- SD (** $p<0.01$ as determined using a two-tailed student T-test).

Supplementary Figure S22. Effect of ectopic AR-V7 and PARP1/2 inhibition on DDR-associated gene expression in LNCaP cells. LNCaP cells transiently transduced with control or AR-V7-expressing lentivirus for 24 hours and then treated with 1 µM talazoparib (Talaz) for an additional 24 hours were subject to quantitative RT-PCR to assess expression of DDR-associated genes. Data represents three independent experiments +/- SD (** $p<0.01$ as determined using a two-tailed student T-test). Genes were segregated into those up-regulated by ectopic AR-V expression and sensitive to PARP inhibitor (top left panel); those enhanced by AR-V7, but insensitive to PARP blockade (top right panel); and those AR-V independent (bottom panel).

Supplementary Tables

Supplementary Table S1 (up-regulated genes in response to AR-V depletion)

AATK	AKR1A1	ABCD1	ACE	ACTL10	ABHD14B	ACBD4	ABCA1
ADAT3	ATP7A	AMOTL1	ANKRD16	ALX4	ABTB1	ACPP	ADM
ATG2A	BDH2	AMPD2	ASTN2	ANKRD52	ADCK5	ADAM15	ALOX15
BAHD1	C4orf48	ARAP2	BAIAP2L2	ANXA9	AMIGO2	ADAMTS1	AMER3
BID	CA11	ARNTL2	BPNT1	APPL2	ANG	AES	ANXA1
C17orf103	CALCOCO1	BICD2	C1orf115	ARSJ	ASS1	ARHGEF3	AQP3
C19orf24	CAMK2N1	BTBD9	CDH7	ATXN7L1	BOK	ATP2B1	B3GNT3
C6orf132	CBX6	C20orf118	CFD	BSPRY	CACFD1	ATP8A1	CDC42EP3
C7orf43	CLN8	C4orf32	CYP1A1	CASP7	CDKN1A	C15orf57	CNTN3
CARD14	CLTB	CAMK1	DBN1	CDIPT	CHRM3	CCDC120	CREB3L1
CARNS1	CRB3	CAMLG	DDN	CDK8	CLSTN3	CPEB3	DSCAM-AS1
CBLC	CREBL2	CTC1	DNAJC18	CHFR	CYB561D1	CYSTM1	ELF3
CHRNE	DNALI1	CTXN1	DSC2	COL5A2	DDHD1	DUSP1	ENPP4
COBL	DUSP2	CUEDC1	EFNA1	DNASE1	DUSP16	DYRK1B	FBLIM1
DBC1	EEF1A2	DACH1	EML6	DOPEY2	EFNB2	EFNA3	FLJ38109
DCAF12L2	EID1	DEGS2	ERBB3	ELMO3	FAM102A	EGR1	GAB2
DEAF1	EPAS1	DHRS3	ERGIC1	EPHB3	GLYCTK	FAM195B	GATA2
DENND5A	EPN3	DOCK4	ERO1L	FAM109A	GMIP	FAM214B	GPR3
DLG5	FGFR3	ENTPD6	FAM63A	FAM111A	HIST3H2A	FLJ20021	HABP4
ETV4	FKRP	ERBB4	FAM86HP	FGD3	HOXC10	GABARAPL1	HGD
EVPL	FOSL2	FAM173A	FNIP2	FXYD3	HSPG2	HIST1H3H	HID1
FAM162A	G3BP2	FERMT3	GADD45G	GRAMD1A	IKZF2	HOXA13	HIST1H2AG
FAM84A	HEXIM1	GPR160	GSTT1	HIST1H2AC	IL17RE	IL17RC	HIST1H2BK
FKBP8	IFI35	GYS1	HES7	HOXC13	ITGA3	IMMP2L	KCND2
FZD4	IFT27	KLC3	ICA1	IRAK2	IZUMO4	IRF7	KIAA1467
GGT1	KDM6B	LOC646862	ID1	ITGA5	JOSD2	LAD1	KIAA1522
GREB1L	KIAA0513	MIR600HG	JMJD7	KHNYN	JUN	LOC113230	KRT8
HIST2H2BE	KLF4	MTHFR	JUP	LOC284578	KDM2A	LOC338758	LANCL3
IGFBP3	KLHL28	MVP	KIF9	LXN	KIAA0922	MAFK	LOC10086267
LAMB2	LDHD	MYOF	LATS2	MAPK15	LLGL2	MAP1S	LOC388692
LITAF	LRFN4	NCOA3	LOC283335	MEX3D	LOC729737	MAPRE2	LRP10
LPPR2	LYPLA2	NGEF	LRSAM1	MICAL1	MAP1LC3A	MNT	MAPK13
MROH6	LZTR1	NRP1	MAN2A2	MXRA8	MMP24	MTSS1L	MST4
MXD4	MANSC1	OSGIN1	MAN2B1	MYH14	NACC2	MYRIP	MUC1
MZF1	MAPKAPK3	PAN3	MFSD6	NKAIN1	NFKBIZ	NCAM2	MYO7A
NADK	MARK1	PANX2	NCK2	NOXA1	PLEKHA2	NR1H2	PCED1B
NUDT22	MC1R	PCDHA10	PCBP4	NPDC1	POMGNT1	PCDHA4	PEG10
PLCD3	MFSD10	PERP	PSMB10	NUDT14	PRRG2	PEX11A	PELI1
PLXNA1	MPZL3	PKP2	PTPRK	PCDH19	RAB30	PLEKHA7	PHLDB3
PODXL2	NAP1L3	PLEKHA6	RBPMS	PCDH9	RUNDC3B	PLEKHB1	PIK3AP1
POLD4	NAPRT1	PNPLA6	REEP2	PCNXL3	SDC4	PLXNA2	PLXNB3
PRAC	NDUFB4	PPP2R2A	RHOC	PDE4B	SEMA3C	PTGS2	PRPH

PRRT3	PDF	PRMT6	S100P	PDLIM2	SGSM3	RAB17	PTPRCAP
PVT1	PHF12	PROM2	SERTAD1	PSD3	SH3GLB2	RENBP	PTRH1
QDPR	PITPNM1	RAB24	SGSM1	PTGFR	SIX2	RHPN1	RALYL
RAB11FIP4	PPP2R5A	RNF11	SH2B1	PVRL4	SLC26A11	SATB1	RDH5
RASEF	PTPN21	SAMD10	SH3BGRL2	RAB3B	SLC7A8	SH3RF1	RELB
RFX3	RILP	SAT1	SIDT2	RBMS2	SLIT1	SLC30A10	RNF208
ROBO1	SLC29A4	SEMA3F	SLC12A6	RIT1	SOX9	SLC40A1	SGK2
RSPH1	SLC41A2	SIGIRR	SLC25A29	RNPEPL1	SPHK2	SPRYD3	SI
S100A11	SLC6A6	SLC52A3	SLC39A13	RWDD2A	STK40	SSH3	SLC17A5
SCAND1	SOWAHB	SLC5A6	SP110	SELM	STX12	SYT7	SLC43A2
SHC4	SYTL1	STARD10	STOM	SNX32	SYNGR2	TLL1	SLITRK5
SLC48A1	TEP1	STBD1	STXBP5	SPRY1	TBX2	TM7SF2	SSTR1
TMEM135	TET3	SYNJ2BP	TJP2	THBS1	TJP3	TNFRSF12A	STON1
TNFRSF21	TINAGL1	TMEM184B	TMC6	TMEM79	TPM4	TNFSF9	TMC4
TRPV3	TMEM8A	TPD52L1	TMEM238	TNK2	TSTD1	TRPM4	TMEM125
TYRO3	WWP2	UNC13B	TRADD	TP53INP2	VASN	TRPS1	TNFSF15
WNT9A	XKR8	WDR45	TTBK2	TULP4	ZNF385B	TTC39A	TRIB1
ZBTB7A	ZDHHC18	ZG16B	UNC45A	ZFP36	ZNF524	ULK1	ZCCHC3
ZFP36L2	ZNF341	ZSCAN16	VGLL4	ZFYVE21	ZNF827	ZFP36L1	ZSWIM4

AATK	AKR1A1	ABCD1	ACE	ACTL10	ABHD14B	ACBD4	ABCA1	APCDD1	AHR
ADAT3	ATP7A	AMOTL1	ANKRD16	ALX4	ABTB1	ACPP	ADM	ATP1B1	ARHGAP32
ATG2A	BDH2	AMPD2	ASTN2	ANKRD52	ADCK5	ADAM15	ALOX15	BAMBI	BASP1
BAHD1	C4orf48	ARAP2	BAIAP2L2	ANXA9	AMIGO2	ADAMTS1	AMER3	BCRP2	C11orf95
BID	CA11	ARNTL2	BPNT1	APPL2	ANG	AES	ANXA1	C19orf21	C8orf4
C17orf103	CALCOCO1	BICD2	C1orf115	ARSJ	ASS1	ARHGEF3	AQP3	C2orf15	C9orf152
C19orf24	CAMK2N1	BTBD9	CDH7	ATXN7L1	BOK	ATP2B1	B3GNT3	CCDC159	CACNA1D
C6orf132	CBX6	C20orf118	CFD	BSPRY	CACFD1	ATP8A1	CDC42EP3	CDH18	CCDC64B
C7orf43	CLN8	C4orf32	CYP1A1	CASP7	CDKN1A	C15orf57	CNTN3	CLDN11	CCDC80
CARD14	CLTB	CAMK1	DBN1	CDIPT	CHRM3	CCDC120	CREB3L1	CLDN4	CDH12
CARNS1	CRB3	CAMLG	DDN	CDK8	CLSTN3	CPEB3	DSCAM-AS1	CYP4F35P	CHST2
CBLC	CREBL2	CTC1	DNAJC18	CHFR	CYB561D1	CYSTM1	ELF3	DCUN1D3	COL14A1
CHRNE	DNALI1	CTXN1	DSC2	COL5A2	DDHD1	DUSP1	ENPP4	DUSP4	CTTNBP2
COBL	DUSP2	CUEDC1	EFNA1	DNASE1	DUSP16	DYRK1B	FBLIM1	FAM110C	CYP1A2
DBC1	EEF1A2	DACH1	EML6	DOPEY2	EFNB2	EFNA3	FLJ38109	FLJ22184	DDIT4L
DCAF12L2	EID1	DEGS2	ERBB3	ELMO3	FAM102A	EGR1	GAB2	FLJ23867	DGKA
DEAF1	EPAS1	DHRS3	ERGIC1	EPHB3	GLYCTK	FAM195B	GATA2	FOXO4	ELF5
DENND5A	EPN3	DOCK4	ERO1L	FAM109A	GMIP	FAM214B	GPR3	FOXP4	ENPP5
DLG5	FGFR3	ENTPD6	FAM63A	FAM111A	HIST3H2A	FLJ20021	HABP4	GDPD1	EVX1
ETV4	FKRP	ERBB4	FAM86HP	FGD3	HOXC10	GABARAPL1	HGD	GPR35	FOS
EVPL	FOSL2	FAM173A	FNIP2	FXYD3	HSPG2	HIST1H3H	HID1	GUSBP1	GALNT3
FAM162A	G3BP2	FERMT3	GADD45G	GRAMD1A	IKZF2	HOXA13	HIST1H2AG	HGF	GSN
FAM84A	HEXIM1	GPR160	GSTT1	HIST1H2AC	IL17RE	IL17RC	HIST1H2BK	HIST1H1C	HMGCS2
FKBP8	IFI35	GYS1	HES7	HOXC13	ITGA3	IMMP2L	KCND2	HIST1H3E	HOXC12

FZD4	IFT27	KLC3	ICA1	IRAK2	IZUMO4	IRF7	KIAA1467	HS6ST2	IER2
GGT1	KDM6B	LOC646862	ID1	ITGA5	JOSD2	LAD1	KIAA1522	IL36RN	KIAA1199
GREB1L	KIAA0513	MIR600HG	JMJD7	KHNYN	JUN	LOC113230	KRT8	INPP5A	KIAA1324
HIST2H2BE	KLF4	MTHFR	JUP	LOC284578	KDM2A	LOC338758	LANCL3	JUNB	LCOR
IGFBP3	KLHL28	MVP	KIF9	LXN	KIAA0922	MAFK	LOC100862671	KCNJ11	LNX1
LAMB2	LDHD	MYOF	LATS2	MAPK15	LLGL2	MAP1S	LOC388692	KCNJ3	LOC286367
LITAF	LRFN4	NCOA3	LOC283335	MEX3D	LOC729737	MAPRE2	LRP10	KIF13B	MAFF
LPPR2	LYPLA2	NGEF	LRSAM1	MICAL1	MAP1LC3A	MNT	MAPK13	KLHL1	MAPK4
MROH6	LZTR1	NRP1	MAN2A2	MXRA8	MMP24	MTSS1L	MST4	LHX9	MFSD4
MXD4	MANSC1	OSGIN1	MAN2B1	MYH14	NACC2	MYRIP	MUC1	LIMA1	NCMAP
MZF1	MAPKAPK3	PAN3	MFSD6	NKAIN1	NFKBIZ	NCAM2	MYO7A	LIPH	NOTCH3
NADK	MARK1	PANX2	NCK2	NOXA1	PLEKHA2	NR1H2	PCED1B	LOC100128770	NOV
NUDT22	MC1R	PCDHA10	PCBP4	NPDC1	POMGNT1	PCDHA4	PEG10	LRRC56	OGFR
PLCD3	MFSD10	PERP	PSMB10	NUDT14	PRRG2	PEX11A	PELI1	MAL2	OSBPL5
PLXNA1	MPZL3	PKP2	PTPRK	PCDH19	RAB30	PLEKHA7	PHLDB3	MESDC2	PAN3-AS1
PODXL2	NAP1L3	PLEKHA6	RBPMS	PCDH9	RUNDC3B	PLEKHB1	PIK3AP1	MESP1	PART1
POLD4	NAPRT1	PNPLA6	REEP2	PCNXL3	SDC4	PLXNA2	PLXNB3	NKPD1	PLA2G4A
PRAC	NDUFB4	PPP2R2A	RHOC	PDE4B	SDA3C	PTGS2	PRPH	PBXIP1	PLA2G4F
PRRT3	PDF	PRMT6	S100P	PDLIM2	SGSM3	RAB17	PTPRCAP	PLA2G16	PPFIA2
PVT1	PHF12	PROM2	SERTAD1	PSD3	SH3GLB2	RENBP	PTRH1	PNCK	RALGAPA2
QDPR	PITPNM1	RAB24	SGSM1	PTGFR	SIX2	RHPN1	RALYL	PNPLA7	REG4
RAB11FIP4	PPP2R5A	RNF11	SH2B1	PVRL4	SLC26A11	SATB1	RDH5	PPP1R3B	SCUBE2
RASEF	PTPN21	SAMD10	SH3BGRL2	RAB3B	SLC7A8	SH3RF1	RELB	PTHLH	SLC5A8
RFX3	RILP	SAT1	SIDT2	RBMS2	SLIT1	SLC30A10	RNF208	QSOX1	SPEF1
ROBO1	SLC29A4	SDA3F	SLC12A6	RIT1	SOX9	SLC40A1	SGK2	RAB25	STAP2
RSPH1	SLC41A2	SIGIRR	SLC25A29	RNPEPL1	SPHK2	SPRYD3	SI	RBM11	SULT2B1
S100A11	SLC6A6	SLC52A3	SLC39A13	RWDD2A	STK40	SSH3	SLC17A5	RNF223	SYT4
SCAND1	SOWAHB	SLC5A6	SP110	SELM	STX12	SYT7	SLC43A2	SLCO5A1	SYTL2
SHC4	SYTL1	STARD10	STOM	SNX32	SYNGR2	TLL1	SLITRK5	SMPDL3B	TLE1
SLC48A1	TEP1	STBD1	STXBP5	SPRY1	TBX2	TM7SF2	SSTR1	TMEM2	TMEM45B
TMEM135	TET3	SYNJ2BP	TJP2	THBS1	TJP3	TNFRSF12A	STON1	TNFRSF11B	TNFRSF19
TNFRSF21	TINAGL1	TMEM184B	TMC6	TMEM79	TPM4	TNFSF9	TMC4	TP53INP1	TSPAN1
TRPV3	TMEM8A	TPD52L1	TMEM238	TNK2	TSTD1	TRPM4	TMEM125	TSPAN12	ZDHHC16
TYRO3	WWP2	UNC13B	TRADD	TP53INP2	VASN	TRPS1	TNFSF15	TSPAN15	ZNF385A
WNT9A	XKR8	WDR45	TTBK2	TULP4	ZNF385B	TTC39A	TRIB1	TUFT1	
ZBTB7A	ZDHHC18	ZG16B	UNC45A	ZFP36	ZNF524	ULK1	ZCCHC3	ULK3	
ZFP36L2	ZNF341	ZSCAN16	VGLL4	ZFYVE21	ZNF827	ZFP36L1	ZSWIM4	ZCCHC24	

Supplementary Table S2 (down-regulated genes in response to AR-V depletion)

AAED1	ACADL	ACBD7	ADORA1	AAK1	AADAT	AFAP1L1	ABHD15	ALDH1L2	ARL1

ACOT7	AP1AR	ADSSL1	AGFG2	AKAP5	ACSF2	ANKIB1	ALDH6A1	ALG12	BMP8B
ACTR3	APOD	ALCAM	AMBRA1	ARHGAP11B	ADAMTS3	BLZF1	ALG10	ALKBH8	C5orf22
ALDH3A2	ARHGEF10	AP1S3	ANKRD32	ATP12A	AKAP12	C10orf118	ARHGEF19	BOD1	C9orf37
ANGPTL4	ARHGEF37	APLN	APOLD1	BMP6	AP3M2	C14orf101	ARSB	BRIX1	CBLL1
AR	ARHGEF6	ATCAY	ATOH8	BRCA2	ARHGAP11A	CCDC71L	B3GALTL	C2CD5	CCDC66
ARMCX4	ARL4C	ATP6V0E1	BCL2L11	C4orf21	ARHGAP19	CCNB2	BDP1	CAMK2D	CCDC90B
AUNIP	ATAD5	BARX1	BLM	CDC6	ARPC5	CENPH	BUB1B	CBWD1	CCNE1
BRMS1L	BARD1	BRIP1	BRCA1	CDH23	ATAD2	CENPK	C7orf60	CCSER2	CDKN1C
C11orf92	C11orf93	C16orf55	C17orf104	CECR6	ATG14	CENPN	CCNA2	CDC20	CDKN3
CAST	C1orf112	C9orf40	C19orf57	CEP152	AVPR1A	CENPQ	CCNB1	CDK17	CENPE
CDC25C	CCNE2	CCDC177	C1QL4	COPS7B	BORA	CEP120	CCNJ	DSEL	CENPF
CHRNA2	CCSAP	CEP63	C5	CTSO	C11orf82	CNN3	CDC40	E2F1	CLIC4
CNOT6	CDC42	CLGN	CASC5	CUL4B	C14orf37	COTL1	CDK19	E2F7	COPS6
CSDA	CENPI	CLOCK	CDC7	DGCR5	C18orf54	CTR9	CEP78	EDN2	COX15
CSDAP1	CIT	CORO1A	CDCA8	DHRS2	C1orf21	DCTN5	CKAP2	FADS2	CPS1
DLX1	CLDND1	CTSL2	CENPJ	EPB41	C7orf63	DONSON	CKAP2L	FAM101B	CSRNP2
DMD	CLSPN	DAPL1	CETN3	EPHB2	CASP8AP2	DPYSL5	CLIP1	FBLN1	DHFR
DOCK8	CNKSR2	DNA2	CNTFR	ERCC6L	CCDC18	FLVCR1-AS1	DEK	FBXO5	DIAPH3
EFEMP1	CROT	DNMT3A	DMC1	EXO1	CDCA7	FOXN2	DMXL2	FEN1	DNAJC27
EFHD1	CRYM	ELL2	DTD1	FAM120C	CDK1	FZD2	EIF5A2	FUT10	DTX4
FKBP5	DCAF12	ESR1	EBAG9	FAM72B	CLIP2	GEN1	ESCO2	GABPB2	DUS4L
FOXD2-AS1	DEPDC1	EXOSC1	EME1	G2E3	CMC2	GINS4	EVA1C	GINS3	DYM
FSTL1	DUSP3	FAM57B	ERI2	GADD45B	CRYBG3	GM2A	FAM136A	GOT1	EIF2AK3
GHRHR	FAM104B	FAM72D	FANCI	GNA13	DGUOK	GNG4	GFPT2	GSTM3	EIF4EBP1
GMPR	FAM72A	FMO4	FKBP9	GNAI2	DYNLT1	HIPK1	HAUS3	HAUS6	FANCD2
GNPDA1	FBXW11	GIPC3	FMNL3	GPLD1	E2F2	HK2	KATNBL1	HJURP	HECTD1
GRIN3A	GABARAP	GLT25D2	GPR137C	GPM6A	EGFR	HSPB11	KCTD9	HLTF	HIVEP2
HPGD	GHR	HOOK1	H19	GSG2	ETNK2	ITPRIP	KIAA0232	HMGB2	HNRNPH3
HSPB6	GPC4	HOPX	HAPLN3	HAS3	FADS1	KDM4A	KIAA1731	HOMER2	IER5L
IL1R1	HOXA4	KIF15	ICAM3	HELLS	FAM120AOS	LOC100144603	KIF18B	IDH1	KIF18A
INMT	IGF1	KNSTRN	ISCA1	HMMR	FAM213B	LOC148709	KIF5C	LIG1	KIF23
KCNC4	KCNMB4	KNTC1	ITGB3BP	KIF14	FAM222B	LRCH1	LDLRAD3	LMNB1	KIF5A
KDELC2	KIF11	KRT19	KCTD12	KIF20B	FANCM	LRP11	LOC284889	LPAR3	LMAN1
KLHL42	KLK3	LRRC40	KIAA1524	KIF24	FAS	MKI67	MAP4K2	MAFB	LOC730101
LAMA3	LINC00467	MCAM	KIF21B	KLF11	FGFR1	MPHOSPH6	MCFD2	MAGOHB	MANF
LIN9	LOC100499405	MCM8	KIF26A	KLF9	FOXD4	MPHOSPH9	MCM6	MBOAT2	MCM4
LPGAT1	MAP2K6	METTL7A	LCLAT1	KLK2	GINS1	MT1E	MELK	METTL4	MDM1
MAOA	MGME1	MMS22L	LPCAT4	LIN52	GPAM	NDRG4	NAE1	MMD	MIPOL1
MAP1B	MIS18A	MTBP	LPL	LOC389831	HSPA5	NEIL3	NDC80	MOB1A	MKL2
MRPL11	MT1X	NEDD4L	LRRCC1	LOC645249	INPP5B	NUP35	NIF3L1	MSH2	MT2A
MYO1D	MYBL1	NUP54	MAD2L1	LOC81691	IRS2	OSBPL3	NUCKS1	MTFR2	NEDD1
NCAPG2	NAMPT	NXPE3	MARCKS	MLF1IP	LMNB2	PKIB	PBRM1	MTHFD2	NES
NEMF	NFATC3	PBK	MASTL	MT1G	LOC100288637	POLA1	PGBD5	NUP155	NUP107
NKX3-1	NID1	PDCD4	MCM10	MYLK	LRR1	POLA2	PIGK	ORC1	OAS3

NME4	OTOP3	PFKFB3	MCM3	NEURL	LRRC3	PREP	PNPLA4	ORC3	OPA1
NPTX1	PALLD	PHF16	MFAP3L	NR2C1	MAML1	PTPLB	PPP6R3	PARP2	PAN2
NPTX2	PREX2	PMP22	MIS18BP1	NUF2	NCAPG	RHNO1	PRR14	PCTP	PDS5B
NSA2	PRIMA1	PNMA2	MND1	ONECUT2	NUP43	RTTN	PTENP1	PDP1	PLK4
NTAN1	PSMG2	PNRC2	MTFR1L	OSGEPL1	OGDHL	SENP1	RWDD2B	PI4K2A	PPP1R3E
PCNA	PTMA	POLE2	PDK4	PABPC4	OIP5-AS1	SFSWAP	SDA4A	POGK	PRIM2
PCNP	PTPRB	PPWD1	PIM1	PARPBP	OSTC	SH3PXD2B	SFN	PPIP5K2	PRPS2
PIGP	RAD54B	PSIMCT-1	PLA2G7	PCDHA11	PLEKHG2	SLC25A24	SLC22A31	PSMD5	RNASE4
PLXND1	RB1	PTCH1	PLEKHF1	PLOD2	RAD51	SMC1A	SLC25A40	PTK2B	SLC10A3
PMEPA1	RIMS3	PTPDC1	POLQ	PSIP1	RFC4	SMC2	SLC35A3	RHOBTB2	SMCHD1
PPAP2A	RRM2	PTPRA	PPDPF	PTDSS1	RFC5	SMNDC1	SMCR7	RMI2	SNHG1
PRICKLE2	SGOL1	RAD54L	PRIM1	RAB11B-AS1	RHOQ	SRD5A3	SNRK	RPP30	SPC25
RGS2	SLC47A1	RNF10	PSTPIP2	RAD1	RIBC2	STK39	SPAG5	RTN4RL1	STAM
RRM1	SMC1B	RTKN2	REEP3	RAD51AP1	RMI1	SUZ12	SPDL1	SASS6	STT3B
SCYL2	SMPDL3A	SBF2-AS1	REEP4	RAD51B	SGOL2	TAPT1	ST7L	SCN8A	TAF5
SGMS2	SRRM4	SEC24A	REEP6	RFC3	SKA3	TEAD1	TCF7	SDC2	THSD4
SLC17A7	SS18	SFXN1	SCG3	RPP14	SLC31A2	TMEM116	TFAP4	SLC38A3	TUBE1
SLC38A4	STEAP2	SLC2A12	SEC61B	RPRD1B	SMIM13	TNFAIP8L1	TMEM209	SMC6	UBTD2
SLC47A2	STRIP2	SRPK1	SIGMAR1	RPS6KB1	SPSB1	TRIM35	TMEM47	STYK1	YBX2
SLCO2A1	SUDS3	STIL	SLC25A33	SIMC1	SYNGR3	TSKU	TP53TG1	TMEM48	ZDHHC8P1
SNAI2	TARP	TBC1D1	SSR1	SMC4	TOP2A	TUSC2	TRANK1	TTC28	ZNF185
ST8SIA6	TEX15	TDH	TBPL1	SYT12	UCHL3	TYMS	TRIP13	UBE2C	ZNF215
STEAP1	TMCC3	TICRR	TMEM14B	THAP6	USP31	WDR17	UBE2T	USO1	ZNF778
STEAP1B	TMED7	TRMT112	TMEM194A	TMEM14C	WDR67	ZBTB1	UHRF1	USP28	ZWILCH
TMPRSS2	TMEM123	TTK	TMEM64	TMPO	WDR76	ZBTB10	USP7	WWTR1	
TMX4	TMEM143	TXNDC16	TMOD2	UBE2E3	ZAK	ZMYM1	XPOT	ZNF273	
TNC	TMEM60	VASH2	TMTC1	WDHD1	ZMIZ1	ZNF114	XRCC2	ZNF326	
UNC5B	TSC22D3	WDR92	TP73	YTHDF3	ZNF217	ZNF318	ZDHHC5	ZNF473	
ZBTB16	ZIC5	ZBTB41	TTL	ZBTB8OS	ZNF704	ZNF519	ZNF280C	ZNF829	
ZNF789	ZNF718	ZNF492	ZNF271	ZNF480	ZRANB3	ZNF596	ZNF530	ZWINT	

Supplementary Table S3

Biological process	No. of genes	% of genes	Fold enrichment	P-value (Hypergeometric test)	Bonferroni method (corrected p-value)
cell division	63	9.81	4.96	1.77393E-26	2.10849E-22
DNA-dependent DNA replication	7	1.09	8.46	1.12367E-05	0.133559243

DNA-dependent DNA replication initiation	17	2.65	13.12	1.05751E-15	1.25696E-11
DNA replication	34	5.30	6.95	1.43037E-19	1.70013E-15
mitotic cell cycle	20	3.12	4.56	1.35163E-08	0.000160655
G1/S transition of mitotic cell cycle	21	3.27	5.61	1.0804E-10	1.28416E-06
regulation of transcription involved in G1/S phase of mitotic cell cycle	11	1.71	13.29	1.10301E-10	1.31104E-06
G2/M transition of mitotic cell cycle	19	2.96	4.06	2.05028E-07	0.002436959

Cell Division

KLHL42; NCAPG2; CDC25C; KIF11; RB1; CCNE2; MIS18A; CCSAP; CEP63; KNSTRN; KNTC1; MAD2L1; CETN3; ITGB3BP; CDCA8; REEP4; CDC7; MASTL; LRRCC1; MIS18BP1; REEP3; CENPJ; ERCC6L; NUF2; CDC6; KIF20B; HELLS; GNAI2; SMC4; KIF14; EPB41; DYNLT1; CDK1; NCAPG; BORA; SKA3; NUP43; CCNB2; SMC2; SMC1A; CCNA2; NDC80; HAUS3; SPAG5; KIF18B; BUB1B; CCNB1; SPDL1; UBE2C; FBXO5; HAUS6; TTC28; CDC20; LIG1; ZWINT; BOD1; SPC25; CENPE; ZWILCH; NEDD1; CCNE1; PDS5B; CENPF;

DNA-dependent DNA replication

POLE2; POLQ; RFC3; WDHD1; RFC5; RFC4; POLA1;

DNA-dependent DNA replication initiation

CCNE2; POLE2; MCM8; MCM10; CDC7; PRIM1; MCM3; CDC6; POLA2; GINS4; POLA1; MCM6; ORC3; ORC1; MCM4; PRIM2; CCNE1;

DNA replication

RRM1; CDC25C; CLSPN; BARD1; RRM2; POLE2; MCM8; TICRR; DNA2; BRIP1; MCM10; BRCA1; CDC7; DTD1; MCM3; BLM; EXO1; RAD1; CDC6; RFC3; RFC5; RMI1; CDK1; RFC4; RHNO1; DONSON; POLA2; POLA1; MCM6; ORC3; FEN1; RMI2; ORC1; MCM4;

Mitotic cell cycle

RRM1; KIF11; CIT; MYBL1; PBK; DNMT3A; KIF15; MASTL; CDC6; WDHD1; SKA3; NDC80; XRCC2; CLIP1; KIF18B; BUB1B; PBRM1; CENPE; TUBE1; CENPF;

G1/S transition of mitotic cell cycle

RB1; CCNE2; POLE2; MCM8; MCM10; CDC7; PRIM1; MCM3; CDC6; RPS6KB1; CUL4B; POLA2; POLA1; MCM6; ORC3; ORC1; MCM4; EIF4EBP1; PRIM2; CCNE1; CDKN3;

Regulation of transcription involved in G1/S phase of mitotic cell cycle

PCNA; RRM2; CDC6; KLF11; TYMS; POLA1; FBXO5; ORC1; E2F1; DHFR; CCNE1;

G2/M transition of mitotic cell cycle

CDC25C; CIT; FBXW11; CEP63; MASTL; CENPJ; CEP152; CDK1; BORA; CCNB2; CCNA2; HAUS3; MELK; CEP78; CCNB1; HAUS6; NES; NEDD1; PLK4;

Supplementary Table S4

Biological process	No. of genes	% of genes	Fold enrichment	P-value (Hypergeometric test)	Bonferroni method (corrected p-value)
DNA synthesis involved in DNA repair	15	2.34	11.91	3.13E-13	3.73E-09
DNA repair	32	4.98	4.23	4.63E-12	5.5E-08
DNA ligation involved in DNA repair	3	0.47	8.35	0.004611	1
DNA damage checkpoint	9	1.40	8.34	6.97E-07	0.008287
DNA damage response, signal transduction by p53 class mediator resulting in cell cycle arrest	9	1.40	4.04	0.000354	1
double-strand break repair via homologous recombination	17	2.65	5.91	2.85E-09	3.38E-05

DNA synthesis involved in DNA repair

BARD1; DNA2; BRIP1; BRCA1; BLM; EXO1; BRCA2; RFC3; RAD51B; RAD51AP1; RMI1; RAD51; POLA1; XRCC2; RMI2;

DNA repair

CLSPN; TEX15; POLE2; RAD54L; TICRR; POLQ; BLM; EXO1; RAD1; PARPBP; RFC3; RAD51B; RAD51AP1; WDHD1; RFC5; CDK1; RFC4; ZRANB3; RAD51; ZBTB1; SMC1A; POLA1; XRCC2; UBE2T; UHRF1; FEN1; PARP2; USP28; MSH2; LIG1; FANCD2; PDS5B;

DNA ligation involved in DNA repair

HMGB2; PARP2; LIG1;

DNA damage checkpoint

CLSPN; CEP63; CLOCK; BRIP1; RAD1; RHNO1; DONSON; USP28; E2F1;

DNA damage response, signal transduction by p53 class mediator resulting in cell cycle arrest

PCNA; CNOT6; CDC25C; CENPJ; CDK1; SFN; CCNB1; E2F7; E2F1;

Double-strand break repair via homologous recombination

AUNIP; RAD54B; MCM8; RAD54L; MMS22L; POLQ; BRCA1; BLM; BRCA2; RAD51B; RAD51AP1; RAD51; GEN1; NUCKS1; XRCC2; FEN1; SMC6;

Supplementary Table S5 (Primers used for qRT-PCR and ChIP)

**Oligo Name**	**Sequence (5’→3’)**
HPRT1 mRNA F	TTGCTTTCCTTGGTCAGGCA
HPRT1 mRNA R	AGCTTGCGACCTTGACCATCT
UBE2C mRNA F	TGCCCTGTATGATGTCAGGA
UBE2C mRNA R	GGGACTATCAATGTTGGGTTCT
PSA mRNA F	GCAGCATTGAACCAGAGGAG
PSA mRNA R	AGAACTGGGGAGGCTTGAG
CCNA2 mRNA F	GAAGACGAGACGGGTTGCA
CCNA2 mRNA R	AGGAGGAACGGTGACATGCT
KLK2 F mRNA	AGCATCGAACCAGAGGAGTTCT
KLK2 R mRNA	TGGAGGCTCACACACCTGAAGA
ATAD2 mRNA F	TGGCACCAGCTGTCATTCAT
ATAD2 mRNA R	AGCTTCACGAATCACCTGGG
FKBP5 mRNA F	CCCCCTATTTTAATCGGAGTAC
FKBP5 mRNA R	TTTTGAAGAGCACAGAACACCCT
TMPRSS2 mRNA F	CTGCTGGATTTCCGGGTG

TMPRSS2 mRNA R	TTCTGAGGTCTTCCCTTTCTCCT
FL-AR mRNA F	AACAGAAGTACCTGTGCGCC
FL-AR mRNA R	TTCAGATTACCAAGTTTCTTCAG
AR exon 3 mRNA F	AACAGAAGTACCTGTGCGCC
AR-V1 mRNA R	TGAGACTCCAAACACCCTCA
AR-V3 mRNA F	AGACGAAGCTTCTGGGTGT
AR-V3 mRNA R	CATGCAGTATGGCTTGGG
AR-V5 mRNA R	CAAAGAATTGTGGGTAGGAAGC
AR-V7 mRNA R	TCAGGGTCTGGTCATTTTGA
AR-V9 mRNA R	GCAAATGTCTCCAAAAAGCAGC
DMC1 mRNA F	AGGTGCCAATGGTTATACCG
DMC1 mRNA R	TTGAAGACACCTGGCTCCTC
XRCC2 mRNA F	TCACCTGTGCATGGTGATATT
XRCC2 mRNA R	TTCCAGGCCACCTTCTGATT
RMI2 mRNA F	GGCAGGGTAGTGATGGCGGAC
RMI2 mRNA R	CCTGAACCACTCCCATCACCAT
BRCA1 mRNA F	CTGAAGACTGCTCAGGGCTATC
BRCA1 mRNA R	AGGGTAGCTGTTAGAAGGCTGG
RAD51AP1 mRNA F	CTTCTGGAAGGCAGTGATGGTG
RAD51AP1 mRNA R	AGAGAAGTCTTCGTCATTATCCTC
RAD54L mRNA F	CCCTTTCTTCCATCACCTCGCT
RAD54L mRNA R	GCCTTAGAGCTGTAACCAGGAG
CHEK1 mRNA F	GTGTCAGAGTCTCCCAGTGGAT
CHEK1 mRNA R	GTTCTGGCTGAGAACTGGAGTAC
EXO1 mRNA F	TCGGATCTCCTAGCTTTTGGCTG
EXO1 mRNA R	AGCTGTCTGCACATTCCTAGCC
NBN1 mRNA F	TCTGTCAGGACGGCAGGAAAGA
NBN1 mRNA R	CACCTCCAAAGACAACTGCGGA
RAD54B mRNA F	GGTGTTGTCCAAGCTCTTAGCG
RAD54B mRNA R	AGCATATCCATGACGCTTACATAC
RAD51C mRNA F	GTGAAACCCTCCGAGCTTAGCA
RAD51C mRNA R	CCTGCTCAAGAAGTTCCAGTGC
ABCF2 mRNA F	GAGGTTTCACTGGGAGCAAGATC
ABCF2 mRNA R	CTGTAGCGTCTTCTCCTTGCTC
CLSPN mRNA F	AAGGAGCGAATTGAACGAG
CLSPN mRNA R	TCTGCAGTGCTTTGGCTG
PCNA mRNA F	GCCATATTGGAGATGCTGT
PCNA mRNA R	TGAGTGTCACCGTTGAAGA
BRCA2 mRNA F	GGCTTCAAAAAGCACTCCAGATG
BRCA2 mRNA R	GGATTCTGTATCTCTTGACGTTCC
RAD21 mRNA F	TCCCCCAGAGGAGCCTCCAA

RAD21 mRNA R	AGCAAGAGCTCGCTGGAGACCA

**Oligo Name**	**Sequence (5'→3')**
PSA Enh F (ChIP)	TGGGACAACTTGCAAACCTG
PSA Enh R (ChIP)	CCAGAGTAGGTCTGTTTTCAATCCA
PSA Prom F (ChIP)	CCTAGATGAAGTCTCCATTGAGCTACA
PSA Prom R (ChIP)	GGGAGGGAGAGCTAGCACTTG
CCNA2 F (ChIP)	TTAGTGAGCTGTCCAGTGACTCAAT
CCNA2 R (ChIP)	CCCATGTATTAAAGTAGCTTCTGTAAACA
KLK2 Prom F (ChIP)	ACCCCTGTTGCTGTTCATCCTG
KLK2 Prom R (ChIP)	CCGCCCTTGCCCTGTTGG
TMPRSS2 Enh F (ChIP)	TGGTCCTGGATGATAAAAAAAGTT
TMPRSS2 Enh R (ChIP)	GACATACGCCCCACAACAGA
UBE2C Enh F (ChIP)	TGCCTCTGAGTAGGAACAGGTAAGT
UBE2C Enh R (ChIP)	TGCTTTTTCCATCATGGCAG

Supplementary Table S6 (siRNA sequences used in study)

**Oligo Name**	**Sequence (5'→3')**
siARex1	CAAGGGAGGUUACACCAAA
siARex4	CCAUCUUUCUGAAUGUCCU
siARex7	GGAACUCGAUCGUAUCAUU
siAR-V	GUAGUUGUGAGUAUCAUGA
siScr	UUCUCCGAACGUGUCACGU

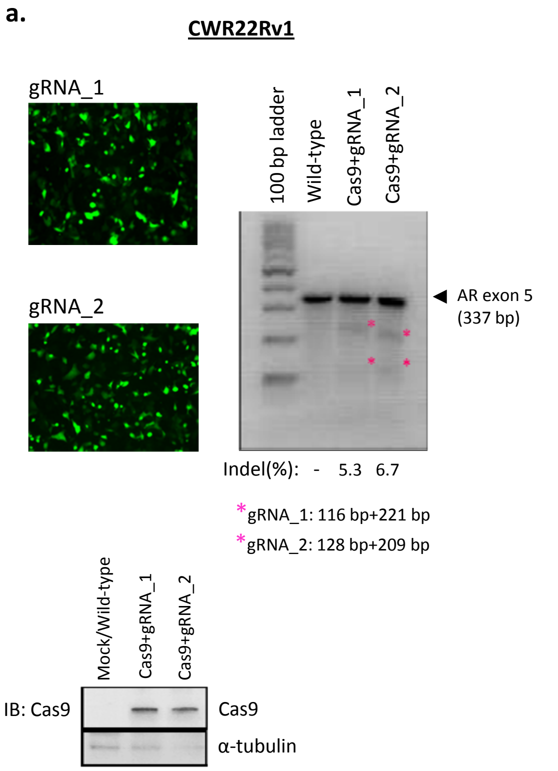

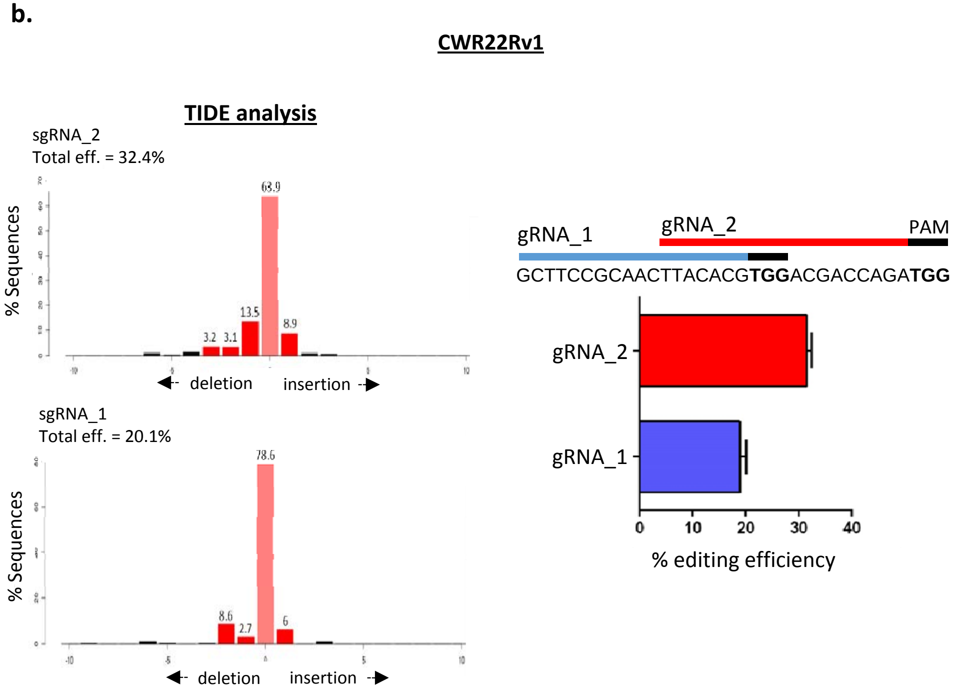

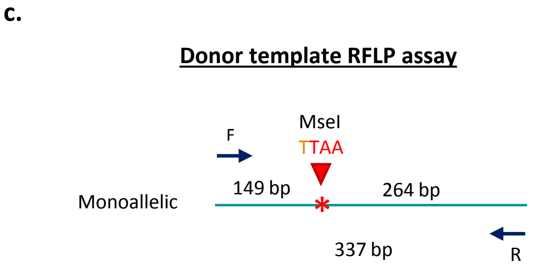

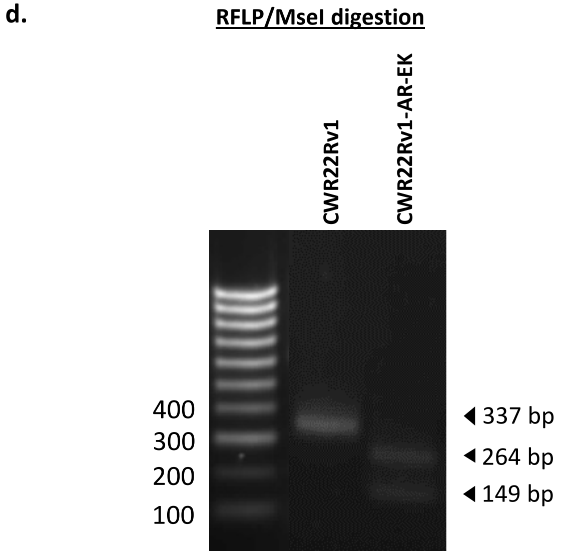

Supplementary Figure S1

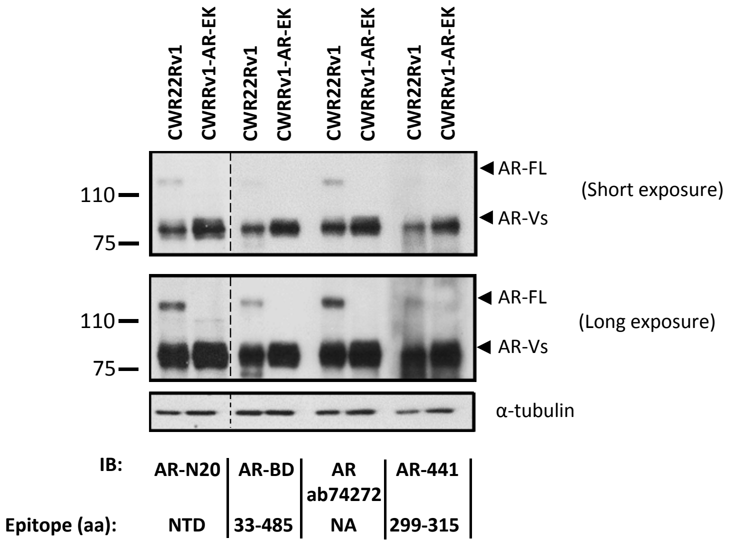

Supplementary Figure S2

a.

CWR22Rv1 | CWR22Rv1-AR-EK

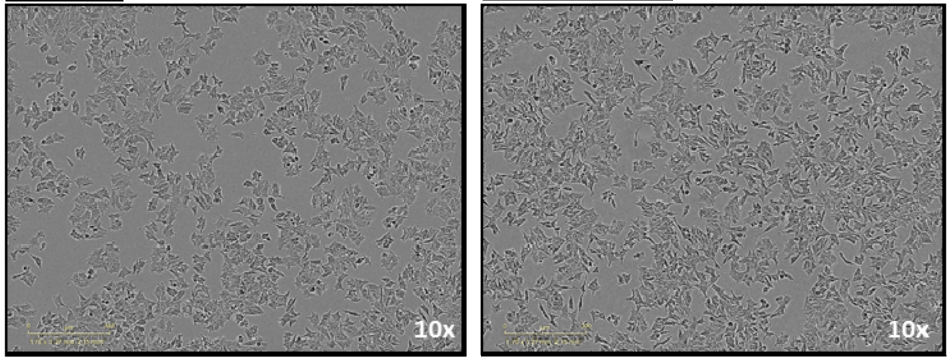

b.

Cell line validation

Markers	CWR22Rv1	CWR22Rv1 AR-EK
Amelogenin	X,Y	X,Y
vWA	15,21	15,21
TPOX	8	8
THO1	6,9.3	6,9.3
D21S11	30	30
D5S818	11,12	11,12
D13S317	9,12	9,12
D7S820	10,11	10,11
D16S539	12	12
CSF1PO	10,11	10,11

c.

CRISPR off-target analysis: CWR22Rv1-AR-EK

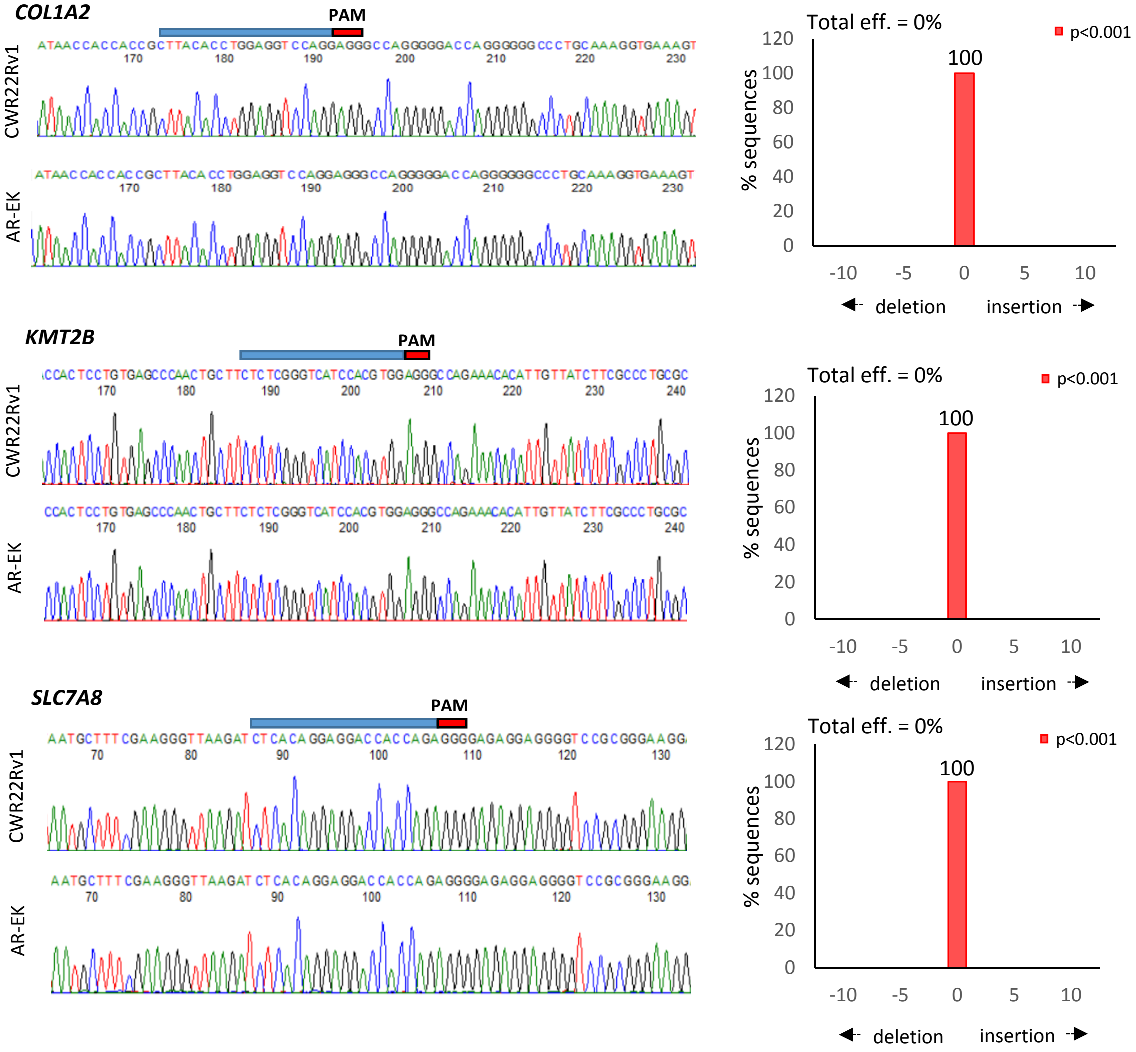

Supplementary Figure S3

CWR22Rv1-AR-EK

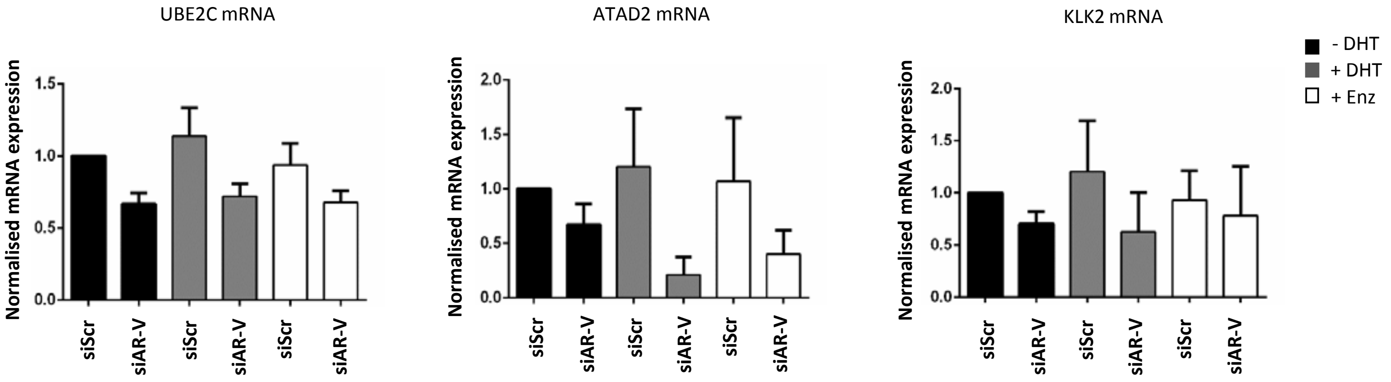

Supplementary Figure S4

CWR22Rv1-AR-EK

a.

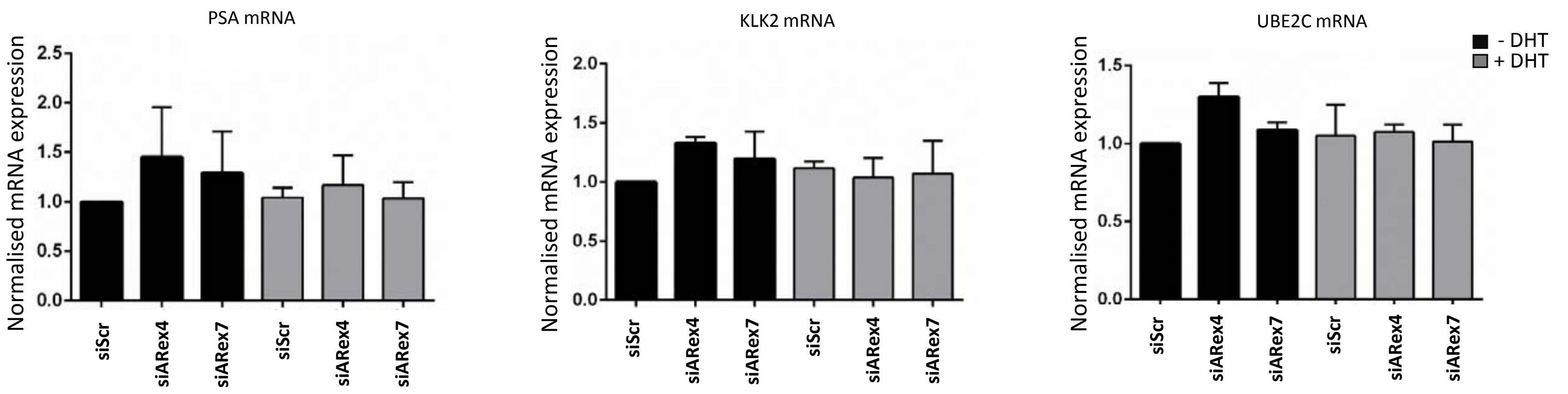

b.

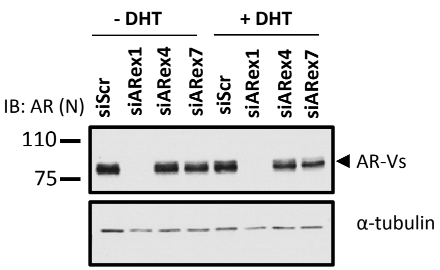

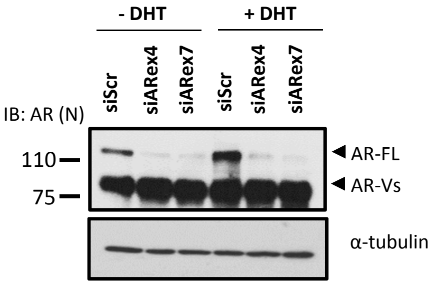

Supplementary Figure S5

CWR22Rv1-AR-EK

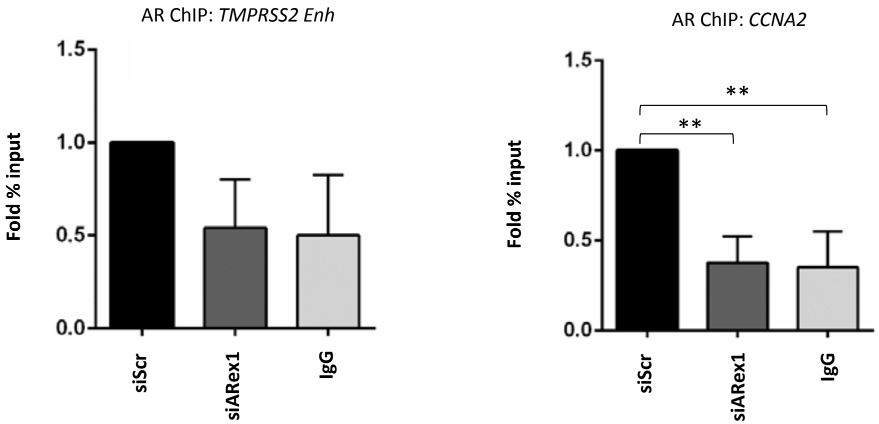

Supplementary Figure S6

a.

CWR22Rv1-AR-EK

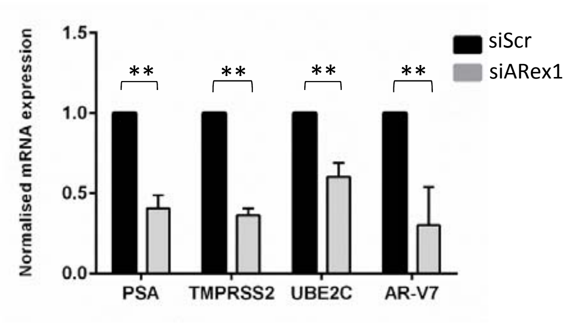

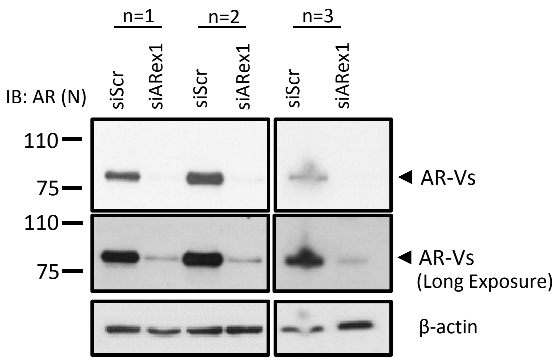

b.

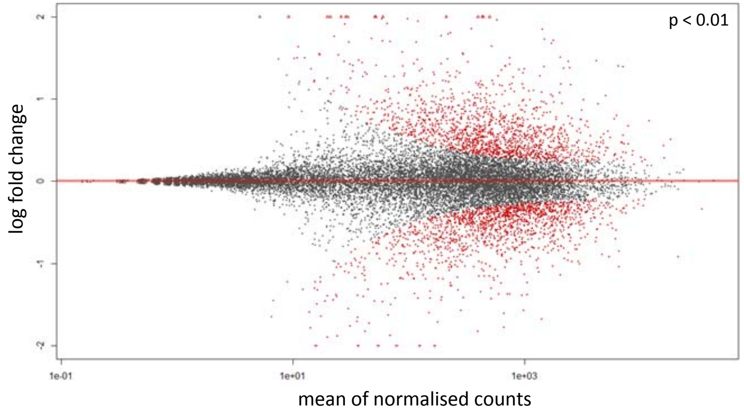

Supplementary Figure S7

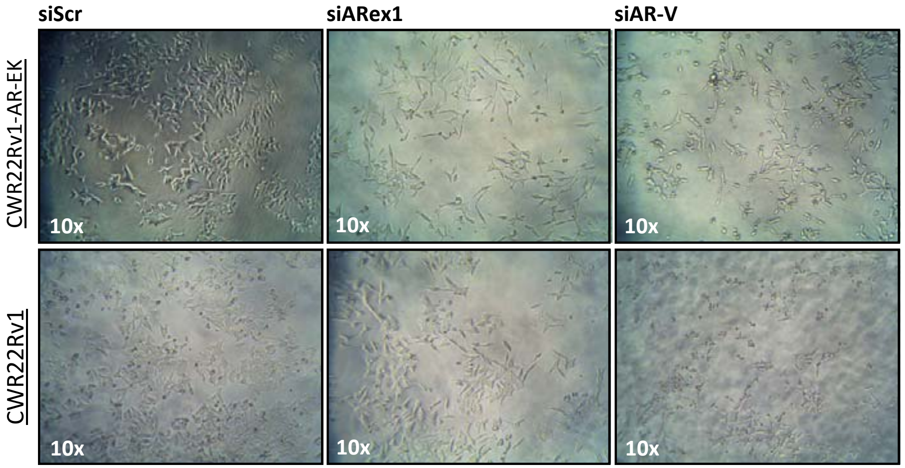

Supplementary Figure S8

CWR22Rv1-AR-EK

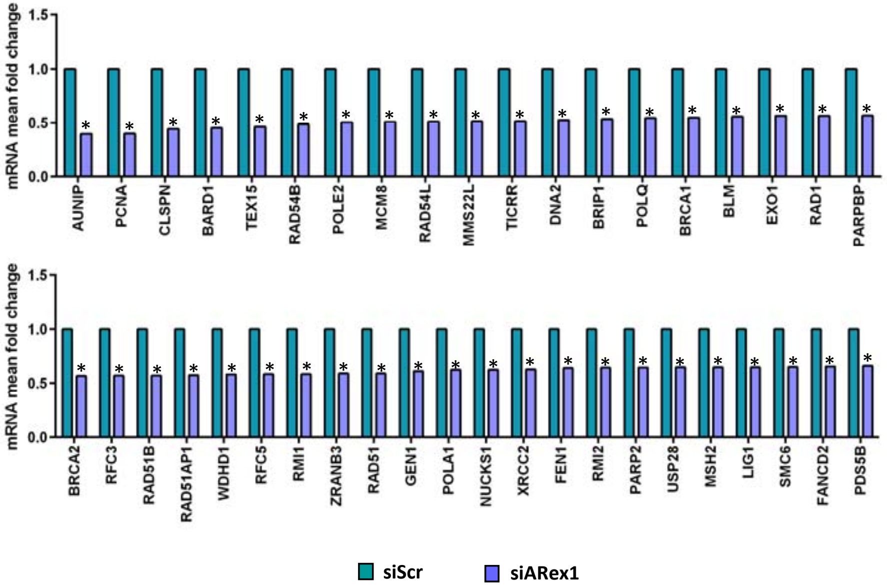

Supplementary Figure S9

CWR22Rv1-AR-EK

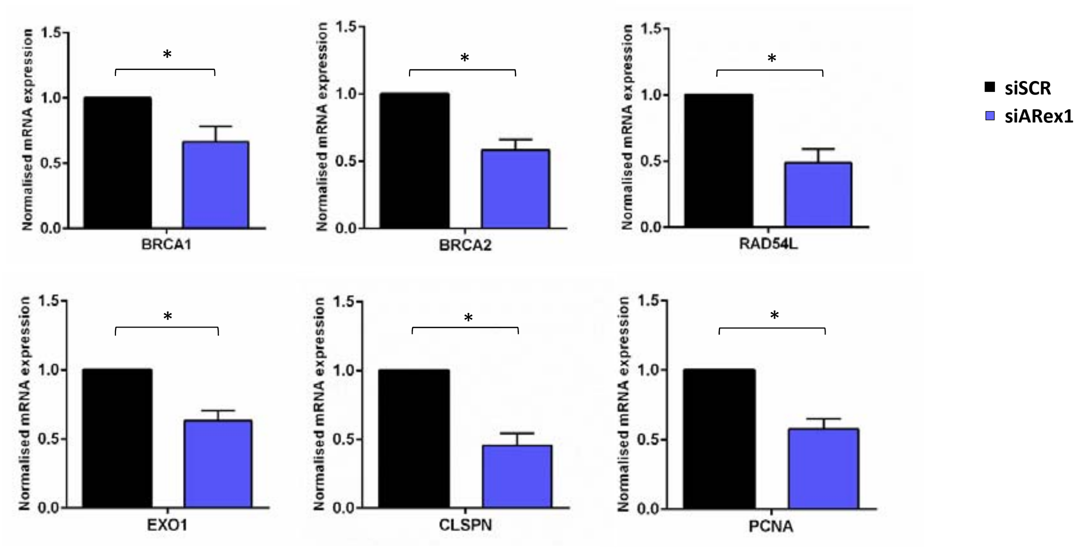

Supplementary Figure S10

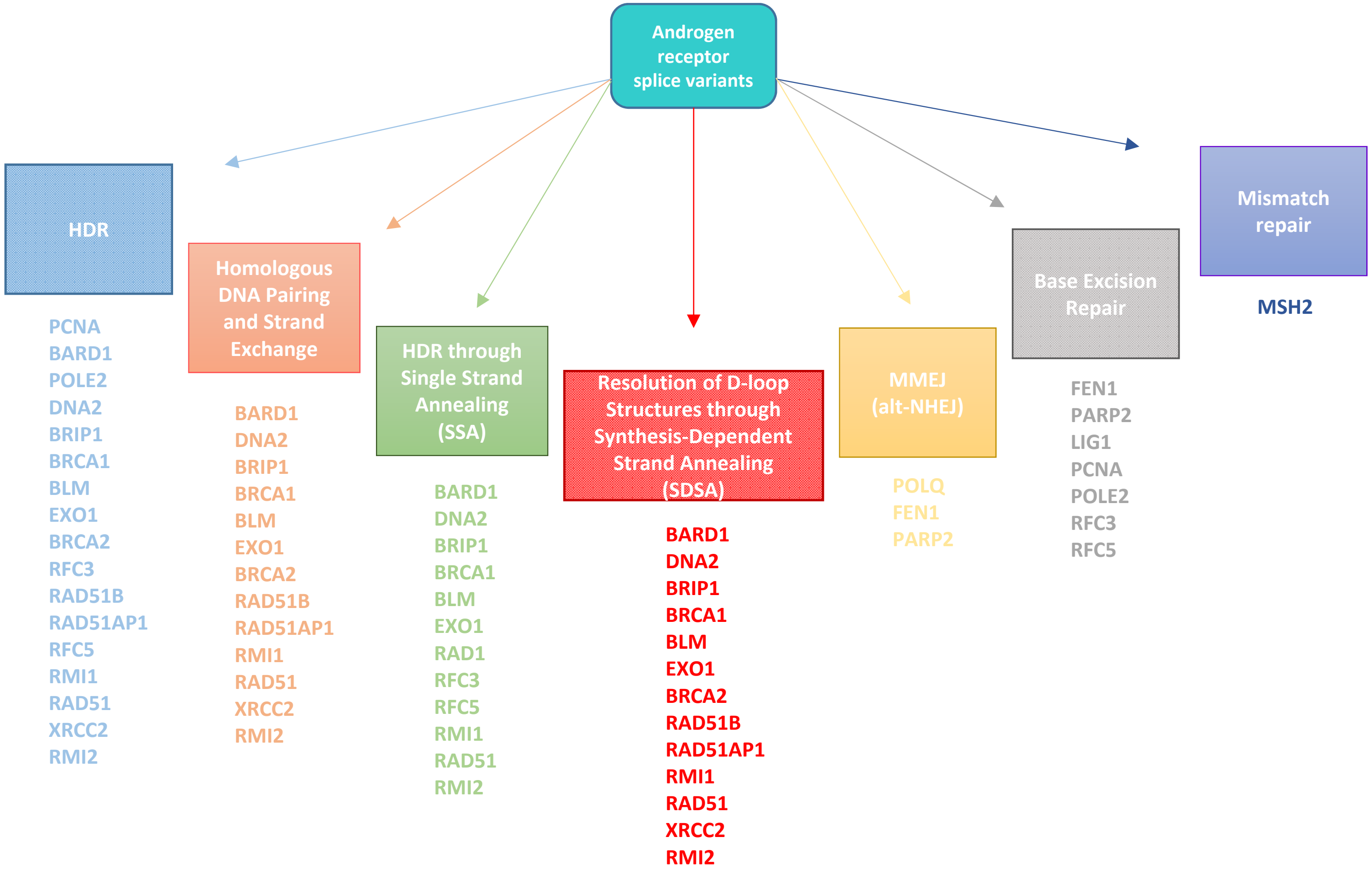

Supplementary Figure S11

Grasso *et al*. 2012

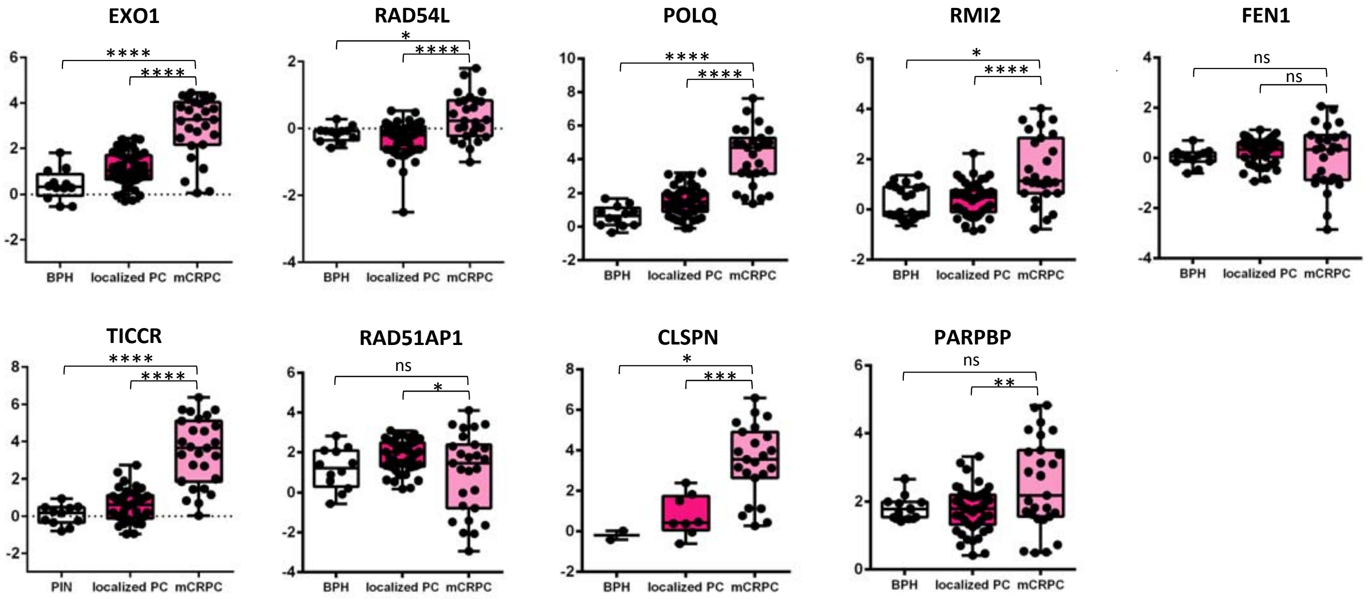

Supplementary Figure S12

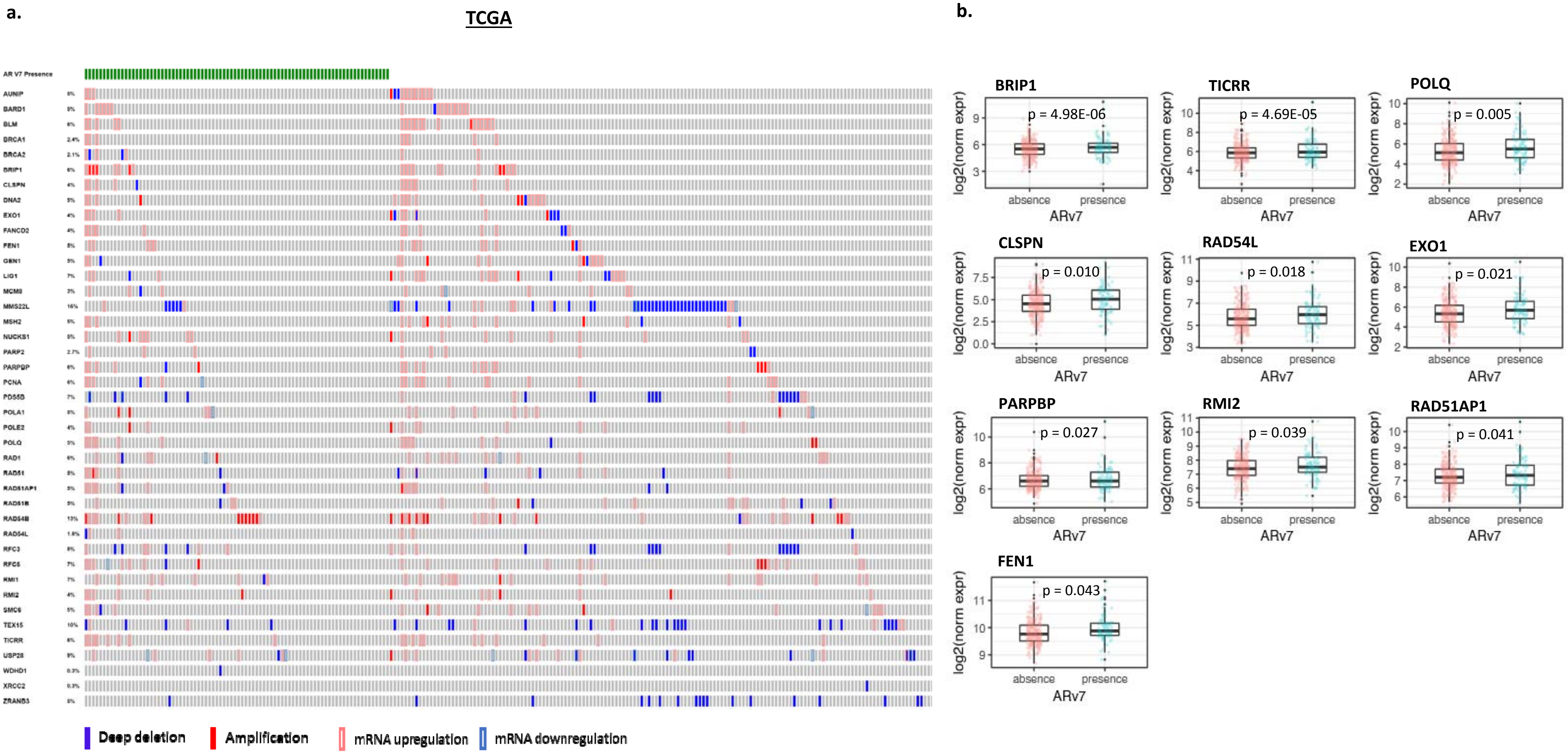

Supplementary Figure S13

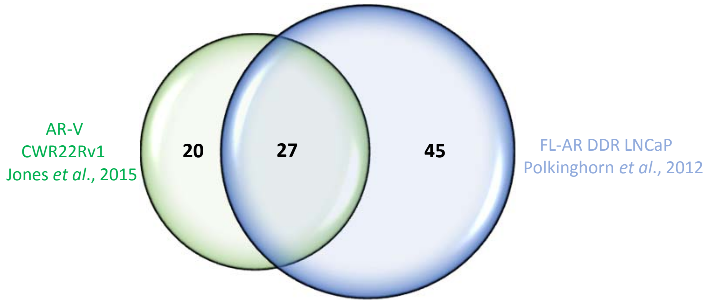

Supplementary Figure S14

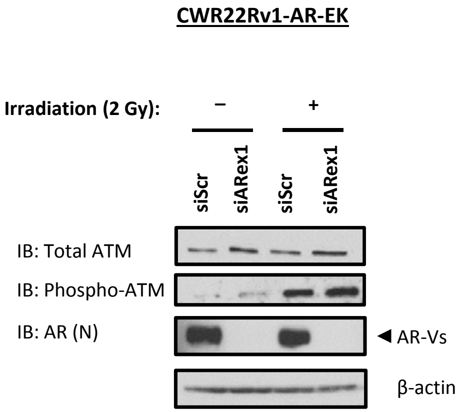

Supplementary Figure S15

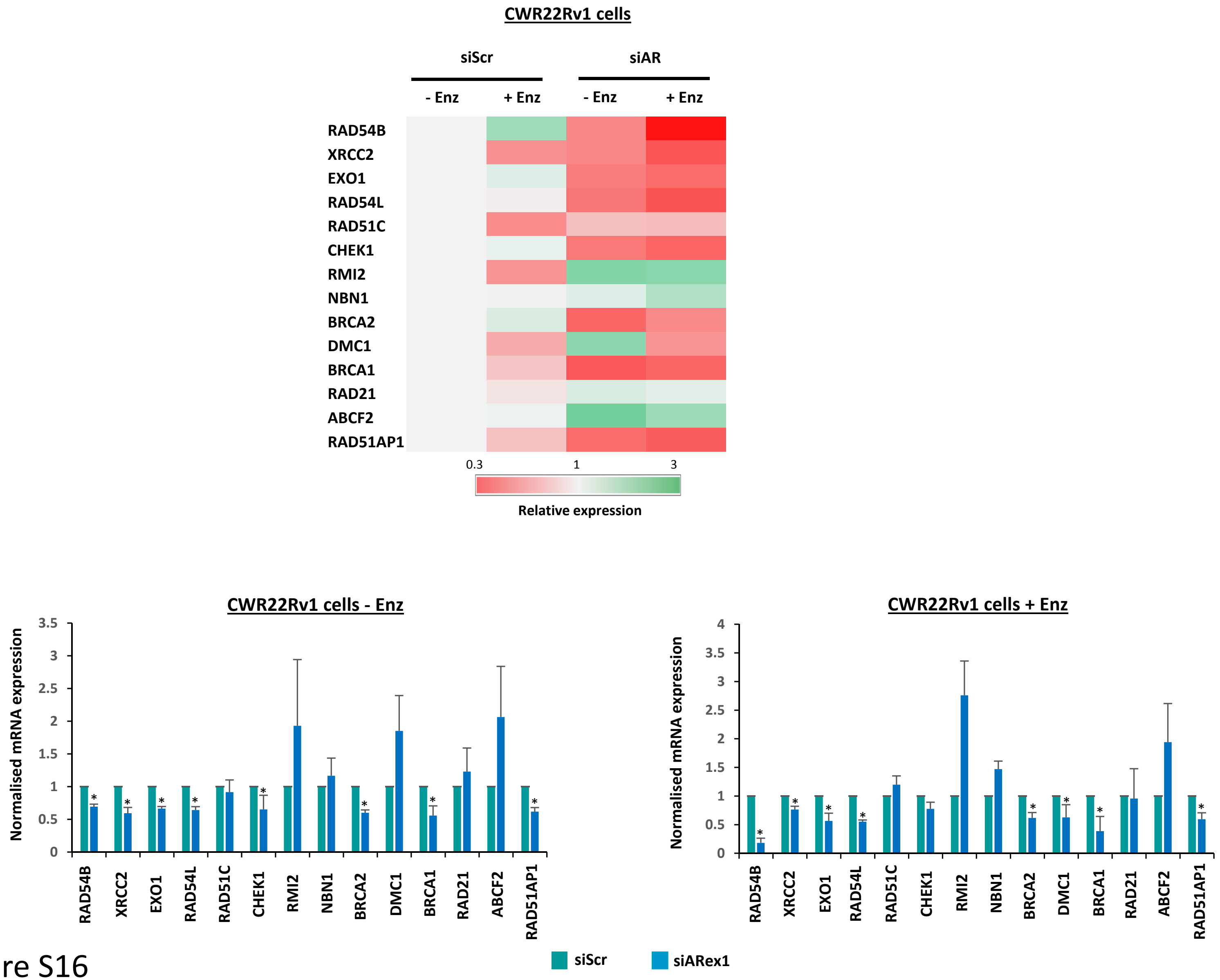

Supplementary Figure S16

CWR22Rv1-AR-EK

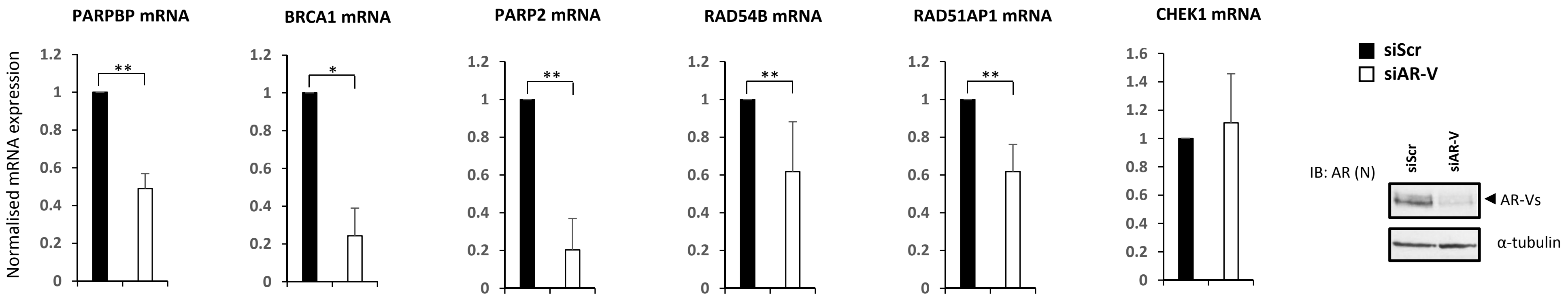

Supplementary Figure S17

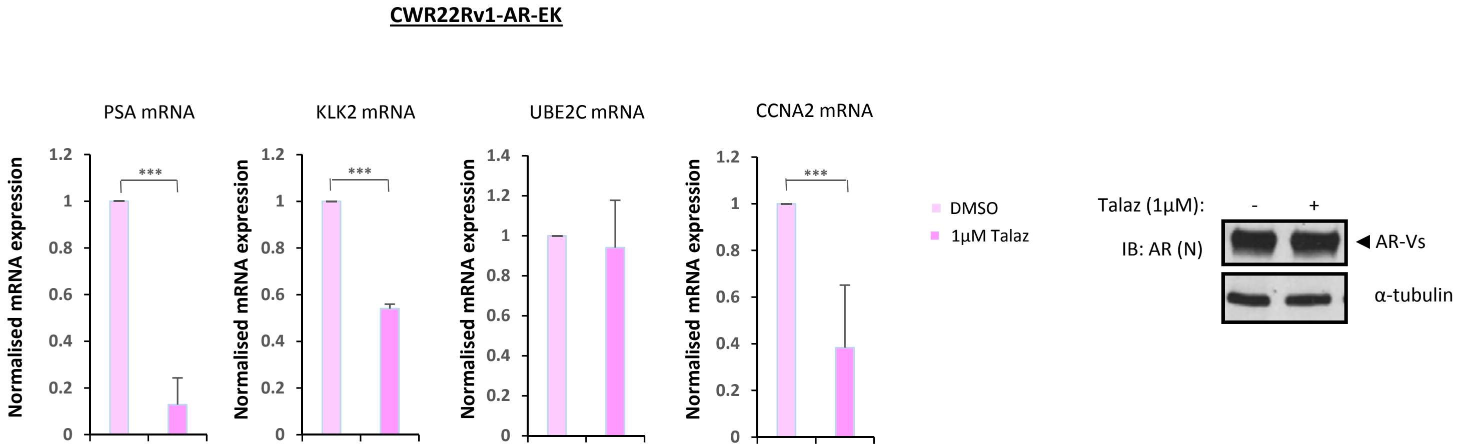

Supplementary Figure S18

a.

CWR22Rv1-AR-EK

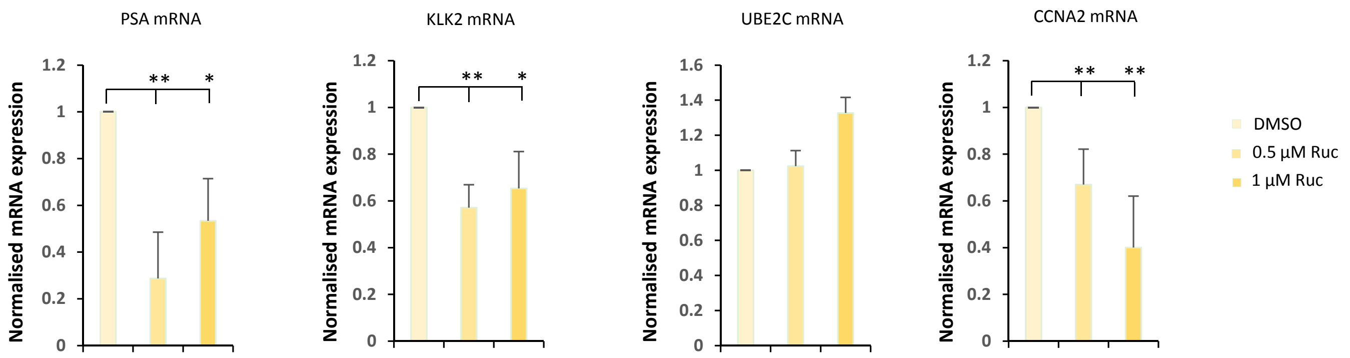

b.

CWR22Rv1

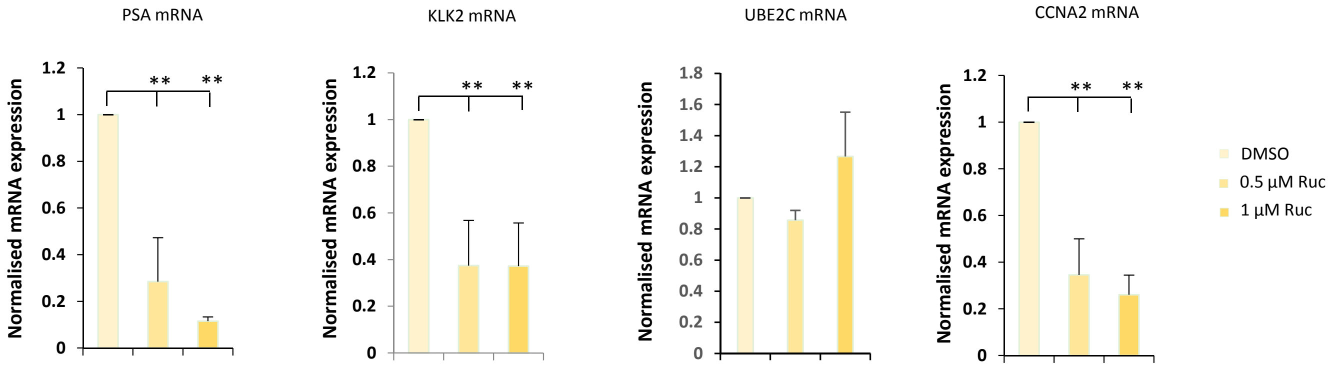

Supplementary Figure S19

a.

CWR22Rv1-AR-EK

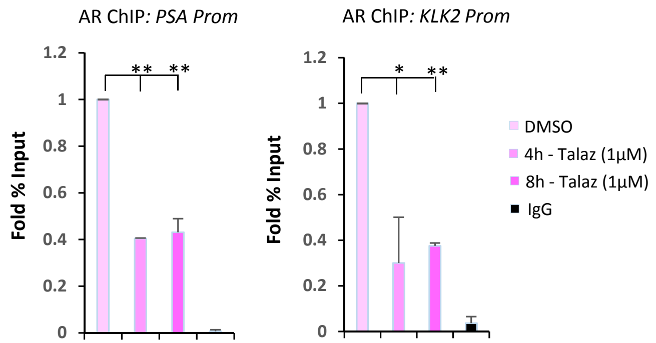

b.

CWR22Rv1

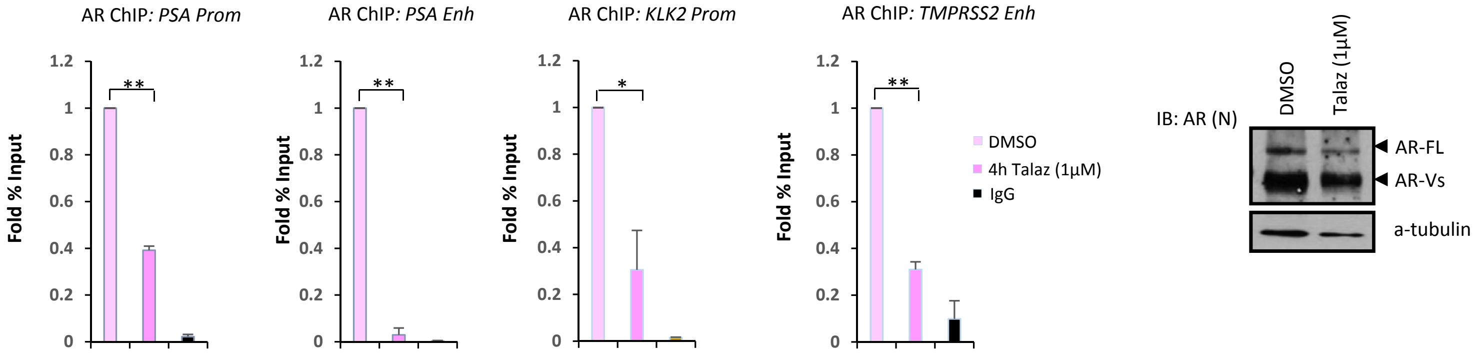

Supplementary Figure S20

CWR22Rv1-AR-EK

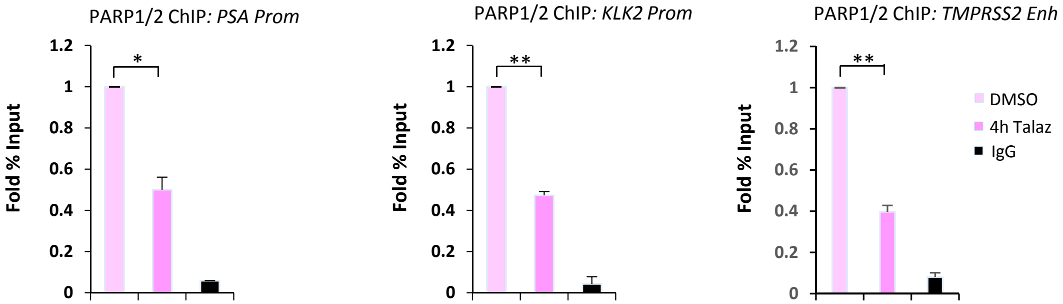

Supplementary Figure S21

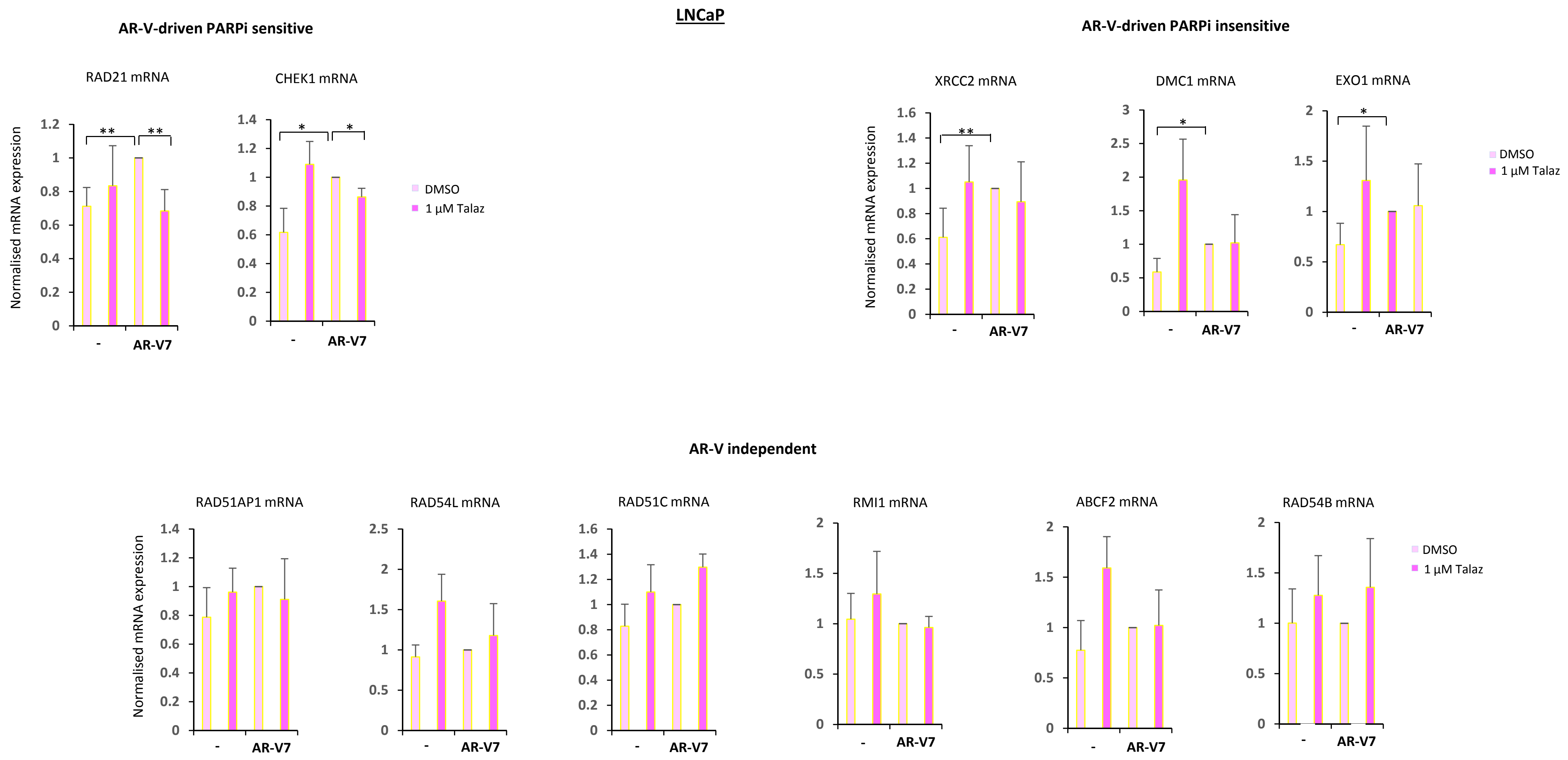

Supplementary Figure S22
